# Early evolution of the biotin-dependent carboxylase family

**DOI:** 10.1186/1471-2148-11-232

**Published:** 2011-08-09

**Authors:** Jonathan Lombard, David Moreira

**Affiliations:** 1 Unité d'Ecologie, Systématique et Evolution, UMR CNRS 8079, Univ. Paris-Sud, 91405 Orsay Cedex, France

## Abstract

**Background:**

Biotin-dependent carboxylases are a diverse family of carboxylating enzymes widespread in the three domains of life, and thus thought to be very ancient. This family includes enzymes that carboxylate acetyl-CoA, propionyl-CoA, methylcrotonyl-CoA, geranyl-CoA, acyl-CoA, pyruvate and urea. They share a common catalytic mechanism involving a biotin carboxylase domain, which fixes a CO_2 _molecule on a biotin carboxyl carrier peptide, and a carboxyl transferase domain, which transfers the CO_2 _moiety to the specific substrate of each enzyme. Despite this overall similarity, biotin-dependent carboxylases from the three domains of life carrying their reaction on different substrates adopt very diverse protein domain arrangements. This has made difficult the resolution of their evolutionary history up to now.

**Results:**

Taking advantage of the availability of a large amount of genomic data, we have carried out phylogenomic analyses to get new insights on the ancient evolution of the biotin-dependent carboxylases. This allowed us to infer the set of enzymes present in the last common ancestor of each domain of life and in the last common ancestor of all living organisms (the cenancestor). Our results suggest that the last common archaeal ancestor had two biotin-dependent carboxylases, whereas the last common bacterial ancestor had three. One of these biotin-dependent carboxylases ancestral to Bacteria most likely belonged to a large family, the CoA-bearing-substrate carboxylases, that we define here according to protein domain composition and phylogenetic analysis. Eukaryotes most likely acquired their biotin-dependent carboxylases through the mitochondrial and plastid endosymbioses as well as from other unknown bacterial donors. Finally, phylogenetic analyses support previous suggestions about the existence of an ancient bifunctional biotin-protein ligase bound to a regulatory transcription factor.

**Conclusions:**

The most parsimonious scenario for the early evolution of the biotin-dependent carboxylases, supported by the study of protein domain composition and phylogenomic analyses, entails that the cenancestor possessed two different carboxylases able to carry out the specific carboxylation of pyruvate and the non-specific carboxylation of several CoA-bearing substrates, respectively. These enzymes may have been able to participate in very diverse metabolic pathways in the cenancestor, such as in ancestral versions of fatty acid biosynthesis, anaplerosis, gluconeogenesis and the autotrophic fixation of CO_2_.

## Background

**Figure 1:**
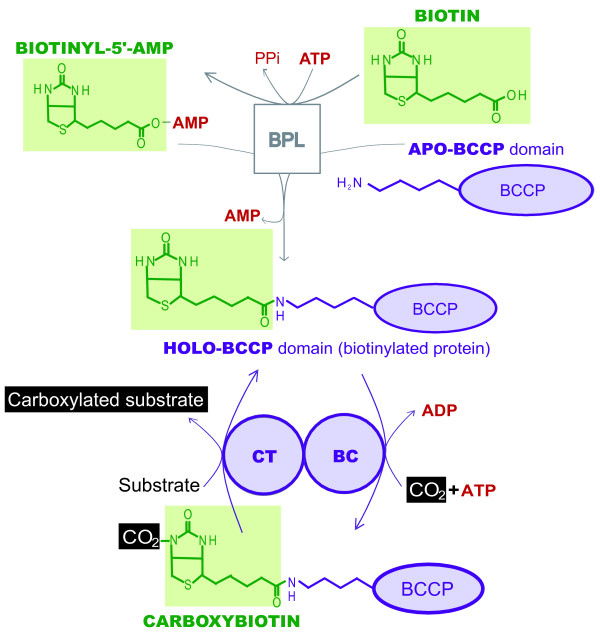
**Schematic diagram showing the reaction catalyzed by the biotin protein ligase (BPL) and the general mechanism shared by the biotin-dependent carboxylases**. Biotin (in green) is first activated to biotinyl-5'-AMP by BPL, then it is transferred by the same enzyme to a biotin carboxyl carrier protein (BCCP). Biotin-dependent carboxylases (in purple) are made up of a biotin carboxylase domain (BC), a carboxyl transferase domain (CT) and the BCCP. The BC domain fixes a CO_2 _molecule to the BCCP-bound biotin and then the CT component binds the carboxyl group to its specific substrate.

Biotin-dependent carboxylases are a group of enzymes present in the three domains of life (Archaea, Bacteria and Eucarya) able to catalyze the fixation of CO_2 _on different specific substrates. They participate in many essential metabolic functions as diverse as the autotrophic fixation of CO_2_, the biosynthesis and degradation of fatty acids, the gluconeogenesis, the anaplerotic production of oxaloacetate or the degradation of some amino acids [1-10]. These enzymes belong to the larger biotin-enzyme family that also contains some biotin-dependent decarboxylases and transcarboxylases [11]. Members of the biotin-enzyme family share functional domains and reaction mechanisms and are characterized by their dependence on covalently bound biotin as a cofactor. Biotin, also called vitamin H, is a prosthetic group made up of a valerate side chain attached to a bicyclic ring consisting of one ureido and one thiophan rings (Figure 1). In this work, we focus on the evolution of the biotin-dependent carboxylases.

The biotin-dependent carboxylase family includes the acetyl-CoA carboxylases, the propionyl-CoA carboxylases, the methylcrotonyl-CoA carboxylases, the geranyl-CoA carboxylases, the acyl-CoA carboxylases, the pyruvate carboxylases and the urea carboxylases. They share a common catalytic mechanism and three functional components: a biotin carboxyl carrier protein (BCCP), a biotin carboxylase (BC) domain and a carboxyl transferase (CT) domain [11]. BCCP is the biotinylated element, which is also shared with the decarboxylases and transcarboxylases of the large biotin-enzyme family. BC catalyzes the ATP-dependent fixation of CO_2 _to the BCCP-bound biotin, and thus the intermediate formation of carboxybiotin. Finally, CT binds the carboxyl group from the carboxybiotin to the specific substrate of each carboxylase (Figure 1).

**Figure 2:**
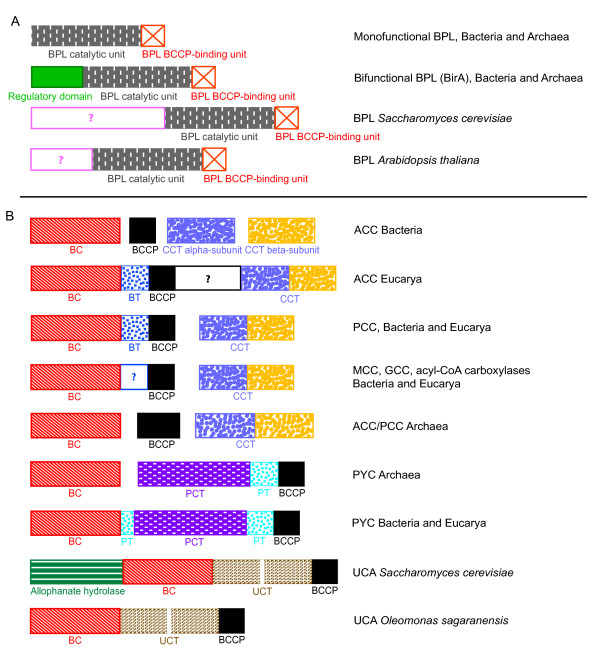
**Protein domain arrangements of different biotin protein ligases and biotin-dependent carboxylases with respect to their functions and phylogenetic origins**. (A) Biotin protein ligases (BPL) and (B) biotin-dependent carboxylases. Homologous protein domains in different enzymes are shown in the same colors and filling. ACC: acetyl-CoA carboxylase; PCC: propionyl-CoA carboxylase; MCC: methylcrotonyl-CoA carboxylase; GCC: geranyl-CoA carboxylase; PYC: pyruvate carboxylase; UCA: urea carboxylase; BC: biotin carboxylase; BCCP: biotin carboxyl carrier protein; CCT: CoA-substrate related carboxyl transferase; PCT: PYC-related carboxyl tranferase; UCT: UCA-related carboxyl tranferase; BT: BC-CT interaction domain; PT: PYC tetramerization.

Before the carboxylation reaction can occur, an independent protein called biotin protein ligase (BPL, also named holocarboxylase synthetase or BirA; E.C. 6.3.4.9; 6.3.4.10; 6.3.4.11; 6.3.4.15) is in charge of the post-transcriptionally attachment of the biotin to a specific conserved lysine residue of the BCCP [12,13]. BPL first adenylates the carboxyl group of the valerate chain of the biotin molecule, then the resulting biotinyl-5'-AMP molecule is used to transfer the biotin moiety to the ε-amino group of the lysine residue of BCCP [14,15]. As each carboxylase has its own BCCP counterpart, it was first thought that each BCCP had one specific BPL devoted to its biotinylation, but subsequent studies have shown that each organism has usually only one BPL protein able to biotinylate the BCCP counterparts from different carboxylases. BPL has even been reported to be able to biotinylate heterologous BCCP elements from other species [12,16,17]. Four types of BPL have been described (Figure 2A, [18]). In prokaryotes, there is one monofunctional sequence bearing only the BPL function [14] and one bifunctional BPL-regulatory gene (BirA). The latter is known to carry, in addition to the BPL catalytic domain, an N-terminus regulatory domain that participates in the transcriptional control of genes involved in biotin biosynthesis [19,20]. Structural comparison and meticulous similarity searches have shown that the BPL biotinylating domain is related to diverse enzymes, including lipoyl protein ligases (LPLs), asparagine synthetases and class II aminoacyl tRNA synthetases [21]. The BPL catalytic domain and the regulatory N-terminal motif have been proposed to have emerged and fused before the radiation of Archaea and Bacteria [22-24]. Concerning the eukaryotes, the N-terminal halves of their BPLs are very diverse: plant BPL contains an N-terminal domain of unknown function and, in contrast with the bacterial N-terminal motif, unable to control the expression of biotin biosynthesis genes [25]. In *Saccharomyces cerevisiae*, a longer N-terminal domain interacting with biotin appears to be involved in the regulation of certain genes (biotin sensing, see Pirner *et al*. [26]).

As previously mentioned, the BCCP domain is characteristic of all the biotin enzyme family, including the decarboxylases and transcarboxylases, whereas the BC domain is limited to the carboxylases. The CT domain gives its substrate specificity to each biotin-dependent carboxylase. Although all biotin-dependent carboxylases bear these three types of protein domains, their arrangement is unequal among different carboxylases and from one domain of life to another. This arrangement will be briefly summarized below (Figure 2B).

Acetyl-CoA carboxylases (ACC; E.C. 6.4.1.2) catalyze the carboxylation of acetyl-CoA to malonyl-CoA in different metabolic processes, such as the first step of fatty acid synthesis or the autotrophic 3-hydroxyproprionate pathway. In *Escherichia coli*, which bears a model bacterial ACC, the three protein domains are encoded by four independent peptides: one BC subunit, one BCCP peptide and two distantly related CT subunits interacting together to ensure the carboxyl transfer function [8]. It is noteworthy that these two distantly related CT subunits are part of a single peptide in all the other biotin-dependent carboxylases bearing them, so here we will refer to the fused peptide as a CT domain and explicitly point to the separation of the CT subunits in bacterial ACC sequences when required. In eukaryotes, the three domains are encoded in a single polypeptide arranged as BC-BCCP-CT from the N- to the C-terminus [1]. Propionyl-CoA carboxylases (PCC; E.C 6.4.1.3) attach one carboxyl group to the propionyl-CoA to synthesize the methylmalonyl-CoA taking part in the 3-hydroxypropionate pathway of CO_2 _fixation, in the synthesis of polyketides and in the degradation of fatty acids and branched-chain amino acids. In bacteria and eukaryotes, PCCs are made up of two polypeptides: a BC-BCCP α-subunit, and a CT-bearing β-subunit. Recent work has shown an additional domain (BC-CT interaction domain, called BT) involved in subunit interactions and located between the BC and the BCCP motifs in the α-subunit [27]. Archaea were thought for a long time to lack ACC and PCC since fatty acids were unknown in this domain of life. Nevertheless, fatty acid synthesis and degradation has been recently detected in archaea and one biotin-dependent carboxylase, using both acetyl-CoA and propionyl-CoA as substrates, has been described in the 3-hydroxyproprionate/4-hydroxybutyrate pathway of CO_2 _fixation of the archaeal order Sulfolobales [6,28,29]. This archaeal ACC/PCC is composed of three different types of subunits bearing respectively BC, BCCP and CT domains.

The 3-methylcrotonyl-CoA carboxylase (MCC; E.C. 6.4.1.4) and the geranyl-CoA carboxylase (GCC; E.C. 6.4.1.5) synthesize 3-methylglutaconyl-CoA and γ-carboxygeranyl-CoA by adding one CO_2 _molecule to their respective substrates. MCC is involved in the breakdown of leucine and acyclic monoterpenes of the citronellol family, whereas GCC appears to participate only in the latter catabolism [9]. While MCC activity has been demonstrated in bacteria and eukaryotes, GCC has only been observed in bacteria. GCC has also been shown to use substrates different from geranyl-CoA [30]. Both MCC and GCC are composed of BC/BCCP-containing α-subunits and CT-bearing β-subunits.

The acyl-CoA carboxylases are a heterogeneous group of enzymes involved in the same pathways as ACC and PCC and also carrying out important functions in the synthesis of secondary metabolites in bacteria. Acyl-CoA carboxylases are basically made up of two types of subunits, the first bearing the BC and the BCCP domains and the second carrying the CT function [7]. As previously shown, this structure is also shared with PCCs, MCCs and GCCs. Indeed, acyl-CoA carboxylases are known to carboxylate acetyl-CoA, propionyl-CoA or butyryl-CoA and, very often, several of these substrates. These promiscuous acyl-CoA carboxylases have been reported in Actinomycetes and Delta-proteobacteria [7,31,32]. Such metabolic promiscuity has led to some confusion in the literature. In the one hand, some authors sometimes consider acyl-CoA carboxylases as PCCs or ACCs according to their preferential substrate, regardless of their possible promiscuous nature or origin [7,31,32]. Moreover, these enzymes can easily change their favorite substrate during evolution, as illustrated by studies showing that the replacement of one single precise residue in the CT sequence of one acyl-CoA carboxylase is enough to shift its substrate specificity [33,34]. In the other hand, most comparative studies on ACC, PCC and MCC activities very often consider each function as being characteristic of one particular carboxylase, ignoring the potential promiscuity typical of these enzymes [11]. Although such functional classifications can be useful to study enzymes within particular metabolic pathways, they may lead to confusion between the well-characterized one-substrate specific carboxylases and promiscuous enzymes carrying out one particular function in a given metabolic context. As an alternative, a phylogenetic approach can complement the functional studies to get new insights on the emergence of functions and classification of this family of enzymes.

Pyruvate carboxylases (PYC; E.C. 6.4.1.1) catalyze the carboxylation of pyruvate to oxaloacetate, an important function for anaplerosis, gluconeogenesis and fatty acid synthesis. A significant feature concerning the protein domain composition of PYC is the homology of the BC and BCCP domains with those of the rest of biotin-dependent carboxylases, in contrast with the independent origin of the CT element. Most bacteria and eukaryotes bear a polypeptidic PYC carrying the BC domain in its N-terminal end, the CT in the central part and the BCCP domain in the C-terminus [35]. Recent studies have discovered a domain situated between the BC and CT domains and between the CT and BCCP domains in *Rhizobium etli *[36-38]. The new domain, referred to as the PYC tetramerization (PT) or allosteric domain, would play a role in tetramerization of PYC subunits and allosteric regulation of PYC by acetyl-CoA. Finally, archaea and some bacteria contain an acetyl-CoA-independent PYC with two different subunits instead of one polypeptide: the BC α-subunit and the CT/BCCP β-subunit [39-42].

Urea carboxylases (UCA; E.C. 6.3.4.6) fix a carboxyl group in urea to form allophanate, an intermediate product of a two-steps process of urea degradation. Allophanate is subsequently hydrolyzed by the allophanate hydrolase to ammonia and CO_2_. While in *Saccharomyces cerevisiae *the allophanate hydrolase and the urea carboxylase are fused within the same polypeptide [43], in the alpha-proteobacterium *Oleomonas sagaranensis *and green algae these two functions are carried out by two independent enzymes homologous to the unique *S. cerevisiae *peptide [44]. The *O. sagaranensis *urea carboxylase is a polypeptide by itself containing a BC domain in its N-terminal end, a BCCP domain in its C-terminus and a predicted central CT domain non-homologous to the previously cited CTs.

These examples illustrate the very complex modular architecture of the biotin-dependent carboxylases, which has been studied for long [45]. This family has been proposed to have evolved by duplication, fusion and recombination events from small monofunctional precursors to produce the modern multifunctional polypeptides [11,45]. The ubiquity of the biotin-dependent carboxylases in the three domains of life and the diversification of their elements have been pointed as arguments for the ancient origin of this family [46,47]. However, hitherto the phylogenetic analyses of biotin-dependent carboxylases have been focused in relatively restricted groups and functions [9,32] or have been carried out with a very limited taxonomic sampling [4,47]. In this work, we attempt to reconstruct the early evolution of the biotin-dependent carboxylases using a global phylogenomic approach in a wide range of taxonomic groups. Our results support previous hypothesis concerning the modular emergence of enzymes of the biotin carboxylase family but also challenge current views on the relationships among different groups of enzymes, especially those related to the acyl-CoA carboxylases. On the basis of phylogenetic evidence, we have tried to define the ancestral content of enzymes from the biotin-dependent carboxylase family in the respective last common ancestor of each domain of life and in the last common ancestor of all living organisms (the cenancestor).

## Results

As mentioned above, the polypeptide composition can largely vary from one biotin-dependent carboxylase to another. Therefore, we have studied each functional protein domain separately. Unlike the biotin carboxylase (BC) domain, which is shared among all the biotin-dependent carboxylases, three non-homologous carboxyl transferase (CT) domains exist in these enzymes: one is common to the ACC, all the PCC, the MCC, the GCC and the acyl-CoA carboxylases and will be called here the CoA-substrate related carboxyl transferase (CCT); another is characteristic of the PYC and will be called the pyruvate carboxylase carboxyl transferase (PCT); finally, UCAs seem to use their own CT (UCT).

### Phylogenetic analysis of the BC domain

The biotin carboxylase domain (BC) is the main distinctive feature shared among all the biotin-dependent carboxylases with respect to the biotin-dependent decarboxylases and transcarboxylases. A critical step in our study was the assignment of each one of the BC domain sequences to their specific function. As the BC domain is a very well conserved motif, it is difficult to distinguish the actual substrate specificity of one precise BC-encoding sequence only on the basis of primary sequence characteristics. Consequently, we constructed preliminary BC domain phylogenetic trees (see Methods and additional file 1) in order to classify each sequence according to a phylogenetic framework. Functional assignment of the bacterial sequences was summarized in a presence-and-absence pattern (Table 1, for complete data see additional file 2).

**Table 1:** Bacterial phyla bearing biotin-dependent carboxylase sequences

	Protein function (protein subunit)										
	
	ACC (BC)	ACC (CT-alpha)	ACC (CT-beta)	PCC (alpha)	PCC (beta)	MCC (alpha)	MCC (beta)	XCC (alpha)	XCC (beta)	PYC	UCA (BC domain)
Alphaproteobacteria (62)	+++	+++	+++	+++	+++	+++	+++	+	+	+++	+
Betaproteobacteria (27)	+++	+++	+++	+++	+++	+++	+++	+++	+++	-	+
Gammaproteobacteria (69)	+++	+++	+++	-	-	+	+	+	+	-	+
Deltaproteobacteria (16)	+++	+++	+++	-	-	-	-	+++	+++	+	-
Epsilonproteobacteria (8)	+++	+++	+++	-	-	-	-	+++	-	-	-
Acidobacteria (3)	+++	+++	+++	-	-	-	-	+++	+++	+	+
Actinobacteria (29)	+	+	+	-	-	-	+++	+++	+++	+++	+
Firmicutes (59)	+++	+++	+++	-	-	-	-	+	+	+++	-
Aquificae (4)	+++	+++	+++	-	-	-	-	+++	-	-	-
Bacteroidetes (20)	+++	+++	+++	-	-	-	-	+++	+++	+	-
Chlamydia/Verrucomicrobia (11)	+++	+++	+++	-	-	-	-	-	-	+	+
Cyanobacteria (18)	+++	+++	+++	-	-	-	-	-	-	-	-
Chloroflexi (5)	+++	+++	+++	-	-	-	+++	+++	+++	-	-
Chlorobi (6)	+++	+++	+++	-	-	-	-	+++	+++	-	-
Planctomycetes (4)	+++	+++	+++	-	-	-	-	-	-	+++	-
Deinococcus-Thermus (3)	+++	+++	+++	-	-	-	-	+	+++	-	-
Spirochaetes (3)	-	-	-	-	-	-	-	+	+	-	-
Thermotogales (5)	-	-	-	-	-	-	-	-	+++	-	-
Fusobacteria (1)*	+++	+++	+++	-	-	-	-	-	+++	-	-
Elusimicrobia (1)*	+++	-	-	-	-	-	-	-	-	-	+++
Dictyoglomi (1) *	+++	+++	+++	-	-	-	-	-	-	-	-
Nitrospira (1)*	+++	+++	+++	-	-	-	-	+++	-	-	-

Numbers next to phyla names correspond to the number of complete genome sequences upon which the search was carried out (see additional file 2 for the complete list). +++, homologues could be found in more than 50% of the genome sequences; +, homologues could be found in less than 50% of the complete genome sequences; -, no homologues found; *, only one genome sequence from these phyla was analyzed.

It is important to note that the BC domain related to the ACC function could be found in almost all bacterial groups, with the exception of Actinobacteria, Spirochaetes and Thermotogales. The PYC polypeptide bearing the BC domain was less widespread but could be found in Alpha- and Delta-proteobacteria, Acidobacteria, Actinobacteria, Firmicutes, Bacteroidetes, Planctomycetes and some organisms from the Chlamydiae-Verrucomicrobia group. Instead of this fused polypeptide, Aquificales and Epsilon-proteobacteria bear an independent PYC-BC domain forming a separate subunit from the CT-BCCP domains. In contrast to the largely distributed ACC and PYC, MCC was limited to Alpha-, Beta- and Gamma-proteobacteria, whereas monofunctional PCC was restricted to Alpha- and Beta-proteobacteria. UCA was found scattered in some representatives of Alpha-, Beta- and Gamma-proteobacteria, of the Chlamydiae-Verrucomicrobia group and of Acidobacteria. Finally, acyl-CoA carboxylases were found to be moderately distributed in all groups of Proteobacteria (except for the Epsilon division), Acidobacteria, Actinobacteria, Bacteroidetes, Chloroflexi, Chlorobi, Deinococcus-Thermus group and one spirochete. In addition to these bacterial sequences, the eukaryotic BC sequences formed well defined functional groups closely related to their bacterial homologues. The only exception to this observation in our preliminary results were the very divergent eukaryotic polypeptidic ACCs, which formed an independent clade probably induced by a long branch attraction artifact due to the acceleration of evolutionary rates subsequent to the polypeptidic fusion of ACC. ACC, PYC, MCC and PCC are widely distributed throughout eukaryotes, while UCA and acyl-CoA carboxylases are limited to fungi and some green algae.

**Figure 3:**
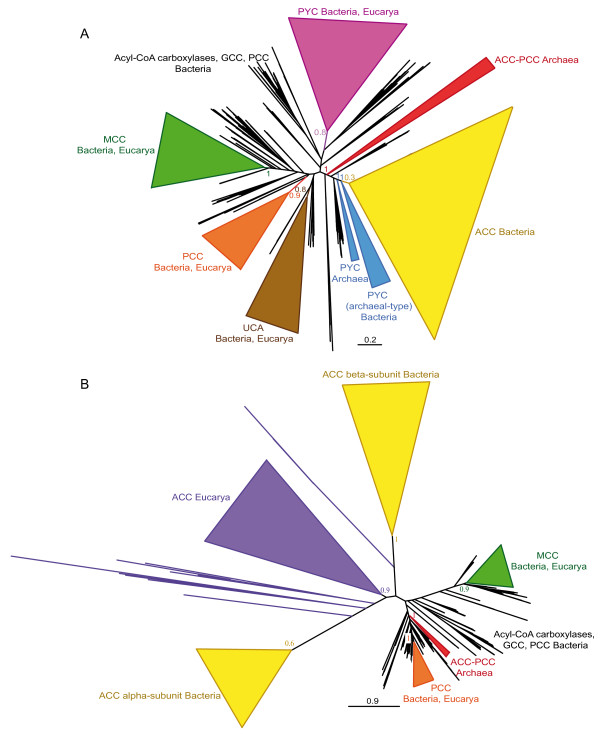
**Maximum likelihood tree of the biotin carboxylase (BC) domain**. This tree is based on 284 representative sequences and 384 conserved sites and was arbitrarily rooted on the bacterial ACC-related sequences. Numbers at nodes indicate bootstrap values higher than 50. Triangles correspond to collapsed groups of eukaryotes and Proteobacteria. Colors on leaves represent the affiliation of the sequences to their respective domain of life: archaea (red), bacteria (blue) and eukaryotes (green). Bars on the right report the functional assignment of the sequences; sequences that are not in front of any bar are assumed to bear an acyl-CoA carboxylase activity.

In order to improve the resolution of the BC domain phylogeny, we selected representative sequences from the complete dataset to carry out maximum likelihood (ML) and Bayesian inference (BI) phylogenetic analyses. The resulting trees were similar to each other, so only the ML tree was shown here (Figure 3, the same holds for the trees of the other protein domains and their respective figures). The BC domain phylogeny confirmed the previous functional classification and supported the monophyly of most bacterial sequences within each functional group. Multifunctional acyl-CoA carboxylase sequences formed different clusters branching at deep nodes of the tree. Eukaryotic sequences clustered again within the groups of their functional bacterial counterparts.

**Figure 4:**
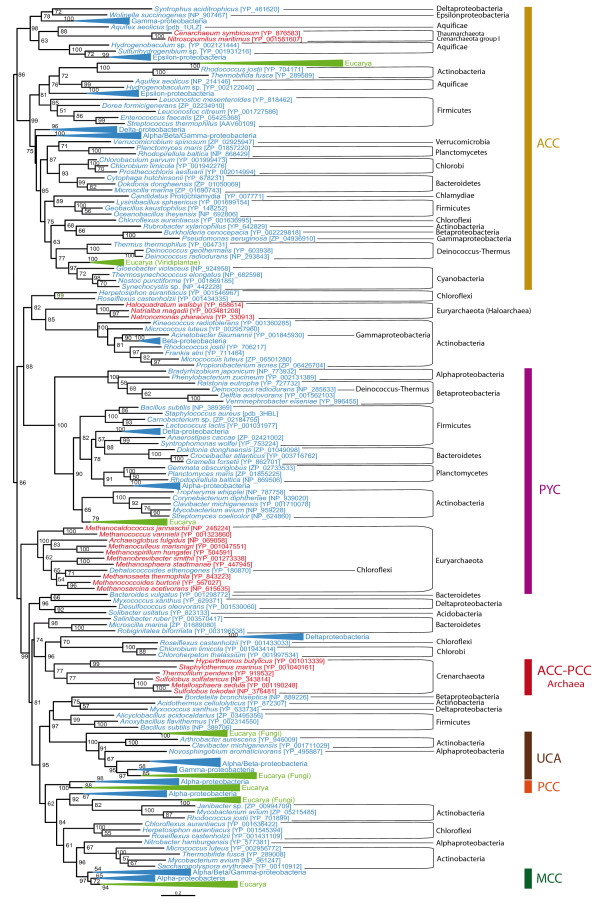
**Maximum likelihood tree of archaeal and bacterial biotin carboxylase (BC) domain sequences**. This tree is based on 196 representative sequences and 322 conserved sites and was arbitrarily rooted on the PYC-related sequences. Numbers at nodes indicate bootstrap robustness values higher than 50. Colors on leaves represent the affiliation of the sequences to their respective domain of life: archaea (red), bacteria (blue) and eukaryotes (green). Bars on the right report the functional assignment of the sequences; sequences that are not in front of any bar are assumed to bear an acyl-CoA carboxylase activity.

In all trees shown above, the archaeal sequences did not form a monophyletic group but were split in several clades within the paraphyletic group of acyl-CoA carboxylases. Nevertheless, these archaeal clades were close to each other, suggesting that they could actually be monophyletic but artificially divided into different clades because of a lack of phylogenetic signal or a phylogenetic reconstruction artifact. As there was little doubt about the position of the eukaryotic sequences with respect to the bacterial ones, we proceeded to the removal of all eukaryotic sequences in order to try to improve the resolution for the prokaryotic sequences, in particular the archaeal ones (Figure 4). As in previous phylogenies, a bacterial ACC clade could be observed in the resulting tree. MCC, UCA and monofunctional-PCC clades branched within a paraphyletic acyl-CoA carboxylase assemblage, and there was a cluster containing all archaeal sequences and bacterial PYCs. Archaeal sequences were not monophyletic but archaeal phyla were retrieved. They occupied closely related basal branches of the PYC cluster with relatively weak support. Consequently, the possibility of an artificial division of archaeal sequences related to a phylogenetic reconstruction artifact could not yet be excluded. We tested this alternative using an unbiased AU test to statically compare the topology shown in Figure 4 with a tree in which the monophyly of archaea was constrained (see Methods). The monophyly of archaea could not be rejected by the test (*P *= 0.44), so archaeal sequences may be monophyletic, as it will be discussed later (see the PCT domain phylogeny section). Interestingly, the BC domain sequences of archaeal ACC-PCC sequences did not cluster among the acyl-CoA carboxylases as could have been expected, but branched among the archaeal PYC sequences. Several bacterial sequences from Aquificales and Epsilon-proteobacteria branched very close to the archaeal sequences, suggesting that they may have acquired these genes by horizontal gene transfer (HGT) from archaeal donors. One representative of these bacterial sequences (*Aquifex aeolicus*) has indeed been deeply studied and shown to adopt an archaeal PYC structure (i.e., two types of subunits instead of one sole polypeptide) and to actually carry out the PYC function [38,42].

### Phylogenetic analysis of the CT domains: The CoA-related carboxyl transferase (CCT)

Since CCT is shared by enzymatic complexes catalyzing carboxylation of different substrates, the first stage of our analysis was to predict the function of each sequence. As for the BC domain analysis described above, a phylogenetic analysis (additional file 1) allowed the functional assignment of CCT sequences based on their position in the resulting phylogenetic tree (results summarized in Table 1, for complete data see additional file 2).

All bacterial species, except the Thermotogales, Spirochaetes and most Actinobacteria, harbored the two CCT-bearing subunits of the bacterial ACC. These independent subunits were very divergent from the rest of the fused CCT domains, probably as a consequence of the separation of the two units in independent peptides. The preliminary phylogenies were congruent with the main bacterial groups within the bacterial ACC clusters. CCT homologues of MCC could be detected in Alpha-, Beta-, and Gamma-proteobacteria, Actinobacteria and Chloroflexi, whereas the CCT domain of monofunctional PCC could only be found in Alpha- and Beta-proteobacteria. A CCT protein domain of acyl-CoA carboxylases (including GCC and likely multifunctional PCCs) could be found in all the proteobacterial groups except the Epsilon-division, in Acidobacteria, Actinobacteria (which possess a high number of paralogues), Firmicutes, Bacteroidetes, Chloroflexi, some Chlorobi, the Deinococcus-Thermus group, Thermotogales and Fusobacteria. Despite a few differences between the BC-domain and the CCT-domain detection (see additional file 2), these results largely matched our previous observations on the presence-absence patterns of the BC domain, supporting the validity of our phylogenetic approach to discriminate sequences according to their functions. Concerning the CCT sequences from eukaryotes, the results from the preliminary phylogenetic reconstructions coincided with those of the BC domain in that all the eukaryotic sequences but the ACC branched in close relationship with their bacterial homologues. As in the BC domain phylogeny, eukaryotic ACC-CCTs formed a highly divergent cluster, what may probably be explained as the result of sequence divergence subsequent to the fusion of different ACC domains into one unique polypeptide.

**Figure 5:**
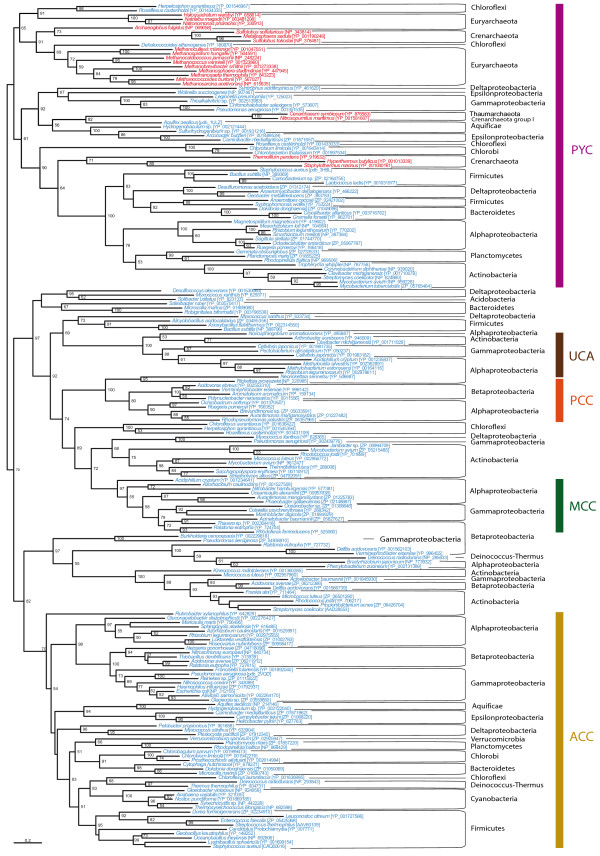
**Maximum likelihood tree of CoA-substrate related carboxyl tranferase (CCT) domain sequences**. This tree is based on 179 representative sequences and 438 conserved sites and was midpoint rooted. Numbers at nodes indicate bootstrap robustness values higher than 50. Triangles correspond to collapsed groups of eukaryotes. Colors on leaves represent the affiliation of the sequences to their respective domain of life: archaea (red), bacteria (blue) and eukaryotes (green). Bars on the right report the functional assignment of the sequences; sequences that are not in front of any bar are assumed to bear an acyl-CoA carboxylase activity.

To improve the resolution of the CCT phylogeny, we selected representative sequences to carry out ML and BI phylogenetic analyses (the CCT subunits of the bacterial ACC and the eukaryotic ACC were removed because of their extreme sequence divergence), with similar results between both techniques (Figure 5). CCT sequences have diverged from each other more than the BC sequences did and, thus, CCT trees are characterized by long branches. Although some HGTs could be identified, major accepted bacterial groups were found within the functional groups described before, suggesting vertical inheritance as a major process to explain their presence in the bacterial genomes. Eukaryotic CCT sequences were also monophyletic within their functional groups and strongly related to their bacterial counterparts.

Finally, although CCT homologues could be detected in a wide diversity of archaeal genomes, we did not retrieve the monophyly of these archaeal sequences in the CCT phylogenies. The statistical support for the separation into several archaeal subgroups was weak, so we tested the possibility that they actually form a monophyletic assemblage by using an unbiased AU test to compare the tree shown in Figure 5 with a tree in which we forced the monophyly of the archaeal sequences (see Methods). The latter could not be rejected by the AU test (*P *= 0.12), opening the possibility that the archaeal sequences may be monophyletic and that the topology observed could be affected by a phylogenetic reconstruction artifact.

### Phylogenetic analysis of the CT domains: The pyruvate carboxylase carboxyl transferase (PCT)

Although the PCT domain is characteristic of PYC and is not shared by any other biotin-dependent carboxylase, the oxaloacetate decarboxylase (ODC; E.C. 4.1.1.3, which takes part in Na^+ ^transport in some bacteria) is also known to use an α-subunit homologous to the PCT domain [48]. Consequently, ODC α-subunit sequences were incorporated into our phylogenetic analyses. In addition, some extremely divergent bacterial homologues showed similarity to transcarboxylases, but we will not treat them here because of their very poor sequence conservation.

**Figure 6:**
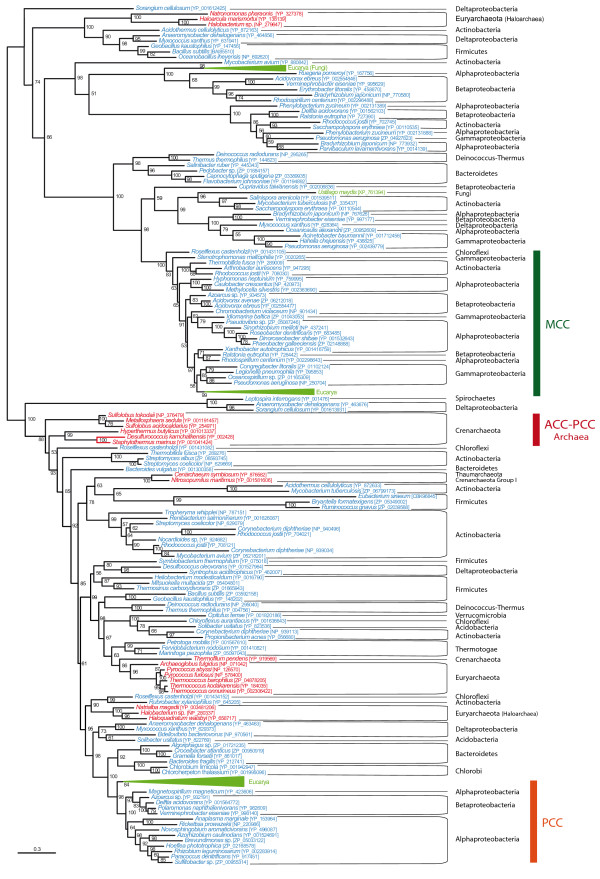
**Maximum likelihood tree of pyruvate carboxylase carboxyl tranferase (PCT) domain sequences**. This tree is based on 126 representative sequences and 432 conserved sites and was midpoint rooted. Numbers at nodes indicate bootstrap robustness values higher than 50. Colors on leaves represent the affiliation of the sequences to their respective domain of life: archaea (red), bacteria (blue) and eukaryotes (green). Bars on the right report the functional assignment of the sequences; sequences that are not in front of any bar have unknown function.

A preliminary tree was used to select a set of representative sequences to reconstruct accurate phylogenies with ML and BI methods (Figure 6). PCT phylogenetic trees showed high divergence between two types of sequences. On the one hand, the PCT domain of bacteria and eukaryotes, which is integrated in their PYC polypeptides. On the other hand, the archaeal-type PCT, found in the β-subunit of the archaeal PYC, in the PCT α-subunit of ODC and in divergent sequences of unknown function in some bacteria, especially in Chlorobi and Epsilon-proteobacteria species. As expected, the PCT domain of the polypeptidic PYC sequences showed the same distribution and phylogeny as the BC domain of the same PYC sequences. In particular, the PCT phylogeny supported the monophyly of the bacterial phyla and the position of the eukaryotic lineage within the bacterial radiation. The phylogeny of the archaeal PCT sequences was also congruent with the accepted archaeal groups. The archaeal cluster contained the bacteria known to carry the archaeal BC-PYC subunit (see above) and some Firmicutes. This result supported an HGT from these archaea to the concerned bacterial groups. Well-characterized ODC homologues are distributed in a limited group of Gamma-proteobacteria and some sporadic bacteria, what supports the restriction of this function to a very small diversity of organisms.

### Phylogenetic analysis of the CT domains: The urea carboxylase carboxyl transferase (UCT)

Compared to the rest of enzymes of the biotin-dependent carboxylase family, little is known about the reaction mechanism and biochemical properties of UCA. Although significant similarities to the BC and BCCP domains have been identified in the UCA gene from the alpha-proteobacterium *Oleomonas sagaranensis*, almost no information exists for the predicted central UCT domain [44]. Similarity searches carried out with this central domain retrieved a protein domain annotated as allophanate hydrolase, probably because the first identified UCA gene was the one of *Saccharomyces cerevisiae*, in which the allophanate hydrolase and the UCA genes are part of a single polypeptide. Thus, the proposed *O. sagaranensis *UCT domain has been annotated as an allophanate hydrolase although it most likely takes part in the UCT function instead of in the allophanate hydrolase.

Fused *S. cerevisiae*-like UCA-allophanate hydrolase polypeptides were found only in some eukaryotes, whereas *O. sagaranensis*-like UCAs containing a BC, a BCCP and the hypothetical UCT domain were detected in some Alpha-, Beta- and Gamma-proteobacteria, some Actinobacteria and some eukaryotes. Finally, our UCT searches led to the finding of two hypothetical homologous genes in some bacterial and archaeal (Thermococcales) genomes. One of these genes was similar to the N-terminal region of the predicted UCT whereas the other was similar to the C-terminal region. These two genes were frequently found adjacent to each other in the concerned genomes, but none of them has been studied so far, and thus, functional data are not available.

### Analysis of the BT/PT domain

As mentioned above, extra protein domains involved in subunit interactions have been recently reported in PYC sequences of *Rhizobium etli, Staphylococcus aureus *and *Homo sapiens *[36-38] and in PCC of *Ruegeria pomeroyi *[27]. In the former, the extra domain was called PT (PYC tetramerization) and was shown to contribute to allosteric regulation in addition to subunit interaction. PT is split in two parts, the first between the BC and the PCT domains, and the second between the PCT and the BCCP domains (Figure 2). In PCC, the protein domain relevant for subunit interaction was called BT (BC-CT interaction domain) and found to be located between the BC and the BCCP domains of the PCC α-subunit. Although the BT and the PT domains shared little primary sequence similarity, structural comparisons revealed striking resemblances concerning above all one helix structure followed by several β-strands. Further structural predictions suggested that the intermediate region between the BC and the BCCP domain of eukaryotic ACC is similar to BT/PT domains [27], supporting that this feature may be widely shared among biotin-dependent carboxylases.

Consequently, we attempted to identify BT/PT-like domains within other biotin-dependent carboxylases. High sequence similarity to BT and PT could only be identified among PCC and PYC sequences, respectively, confirming previous results [27,38]. In addition, we observed a protein region of 100-140 residues flanked by BC and BCCP domains in α-subunits from MCC, GCC and acyl-CoA carboxylases (reported in Figure 2 as domains of unknown function). We used two different algorithms (see Methods) to carry out secondary structure searches on the linkers of the MCC, GCC, archaeal and bacterial PYC and acyl-CoA carboxylase sequences. The helix structure characteristic of the BT function in PCC [27] was predicted in all linkers tested, except the acyl-CoA carboxylase characterized by Rodriguez *et al*. [7], for which the two methods applied were not consistent with each other. This enzyme is known to use a supplementary subunit called accE that is involved in subunit interaction [49]. Interestingly, a helix structure was detected in this accE that might carry out the same function as in the BT/PT domain.

### Phylogenetic analysis of the BPL-BirA genes

The biotin carboxyl carrier protein (BCCP) domain is a short biotinylated peptide (~ 90 amino acids) often fused to the other constitutive domains of the biotin-dependent carboxylases. Its short size and participation in very different polypeptide architectures make difficult to reconstruct accurate phylogenies using the BCCP sequences. Consequently, instead of analyzing the short BCCP domain itself, we decided to study the phylogeny of the biotin protein ligase (BPL) responsible of its biotinylation.

**Figure 7:**
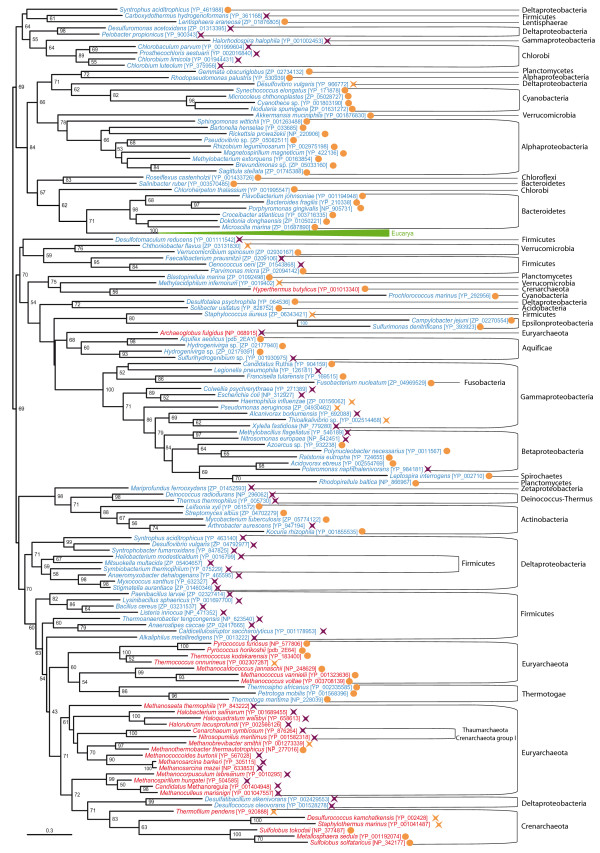
**Maximum likelihood tree of the biotin carboxyl ligase (BPL)**. This tree is based on 156 representative sequences and 164 conserved sites and was arbitrarily rooted. Numbers at nodes indicate bootstrap robustness values higher than 50. Triangles correspond to collapsed groups of eukaryotes. Colors on leaves represent the affiliation of the sequences to their respective domain of life: archaea (red), bacteria (blue) and eukaryotes (green). Purple crosses correspond to sequences bearing a complete N-terminal domain and thus assumed to carry out a BirA regulatory function in addition to the BPL activity; orange circles correspond to sequences lacking the N-terminal domain and thus assumed to carry only the BPL function; orange crosses correspond to sequences that bear an N-terminal domain but lacking conserved positions important for the regulatory function.

In eukaryotes, BPL occurs in a polypeptide in which the C-terminal end bears the BPL function, whereas little is known about the function of the very diverse N-terminal region. As previously mentioned, two main types of BPL can be found in prokaryotes: the so-called BPL that solely bears a BPL function, and BirA, that has a DNA-binding domain in its N-terminus responsible for a transcriptional regulatory role. Although quaternary structure differences among different taxa have been major concerns in previous literature [23,24,50,51], we will only consider here the major distinction concerning the bifunctionality of BirA with respect to the monofunctionality of BPL [22]. Accordingly, we classified prokaryotic sequences into the BPL or BirA groups on the basis of the absence or presence of the DNA-binding N-terminal domain, respectively. Noteworthy, we observed that several BirA sequences lacked the conserved positions important for the regulatory function as established by Mukhopadhyay *et al*. [18]. Except for some rare cases that had already been reported (*Clostridium acetobutylicum, Lactococcus lactis, Halobacterium *sp., *Pyrococcus abyssii, Pyrococcus furiosus*), our BPL-BirA searches confirmed the presence of only one BPL homologue in each prokaryotic genome. The BPL protein was widespread in Archaea, Bacteria and Eucarya and its phylogeny was congruent with the accepted main taxonomic groups within each domain (Figure 7), supporting that an ancient BPL function existed in the three domains of life. Nevertheless, instead of finding monophyletic groups containing uniform functional clades, our results showed the monofunctional and the bifunctional sequences as being mixed up all over the prokaryotic part of the tree whatever the reconstruction method or representative sequence selection dataset was used. Some species bearing both BPL and BirA genes, as *Halobacterium *sp. and *C. acetobutylicum*, had their respective BPL close to their own BirA in phylogenetic trees, whereas *P. abyssii, P. furiosus *and *L. lactis *BPL and BirA genes branched very far from each other.

## Discussion

The large distribution of biotin-dependent carboxylases and their remarkable mechanistic conservation has been interpreted as an evidence for the ancient origin of this enzyme family [46,47]. However, phylogenetic analyses of biotin-dependent carboxylases carried so far were either restricted to some functions and taxonomic groups or used very limited taxonomic sampling [4,9,32,47]. For the first time, here we studied the early evolution of the biotin-dependent carboxylases using a global phylogenomic approach in a wide range of taxonomic groups. Our results support the ancient origin of this family and give new insights on the evolutionary history of these protein domains and enzymes. In particular, we have observed that the domains of life are different with regard to the importance of the biotin-dependent carboxylases in their metabolism and evolution. Consequently, we will first discuss the evolution of these enzymes in each domain of life, prior to conclude on how our results provide information about the biotin-dependent carboxylase content in the cenancestor.

### Biotin-dependent carboxylases in Bacteria

The high diversity of biotin-dependent carboxylase paralogues found in the bacterial domain reflects the major role of these enzymes in bacterial metabolism. The presence-absence study combined to the BC and CCT phylogenies supported the wide distribution and monophyly of bacterial ACC, suggesting that it is ancestral to this domain of life. Similarly, the PCT phylogeny suggested that the polypeptidic PYC is monophyletic and widespread enough to support the ancestral presence of this carboxylase in Bacteria. In contrast, the BC domain devoted to UCA activity is limited to a restricted number of bacteria branching within the acyl-CoA carboxylases from which it probably emerged. Proteins homologous to UCT are distributed among a wider range of bacteria, but these are hypothetical genes and their function is unknown. In order to explain why the distribution of UCT is larger than that of the UCA-related BC, two possibilities exist: either UCT could be specific of biotin-dependent carboxylation and collaborate with other BCs to specifically carry out this function, or the UCT domain could have been recruited from another biochemical reaction, so that certain UCT homologues that we can detect would be representatives of the original function. Thus, there is not enough information to advance a confident hypothesis on UCAs origins, but available evidence could hardly support an ancient origin of UCA in Bacteria and rather suggests its recent emergence and spread by frequent HGT among bacteria.

Sequences annotated as acyl-CoA carboxylases were found to be paraphyletic in BC and CCT phylogenies (Figures 3, 4 and 5). Duplication and HGT events have undoubtedly been of major importance in their evolution, as paralogues and xenologues could be detected in our analyses. Nevertheless, the acyl-CoA carboxylases were distributed in a wide range of bacteria and most often clustered into clades congruent with the accepted main bacterial groups. Moreover, several arguments supported that GCC, MCC and PCC are parts of the acyl-CoA carboxylases: i) GCC, MCC and PCC sequences branched among sequences previously annotated as acyl-CoA carboxylases in our BC and CCT domain phylogenetic trees (Figures 3, 4 and 5); ii) MCCs and monofunctional PCCs were restricted to some proteobacterial groups, supporting the emergence of MCC and PCC functions from an original acyl-CoA carboxylase in a proteobacterial ancestor; iii) the GCC, MCC and PCC sequences shared a common domain architecture with the acyl-CoA carboxylases (Figure 2); iv) some GCC and PCC have been reported to carry out the carboxylation of different substrates, a promiscuity which is characteristic of the acyl-CoA carboxylases [7,30,32]. As a result, our phylogeny-based classification of GCC, MCC and PCC sequences into the acyl-CoA carboxylase group is reliable and strongly supports that these functions are specific adaptations from a promiscuous acyl-CoA carboxylase-like protein according to the metabolic needs of each species, a common process in enzyme evolution [52,53]. Indeed, functional flexibility of acyl-CoA carboxylases may be of major importance in relatively rapid adaptation strategies concerning bacterial metabolism [5,54,55] since minor precise sequence modifications have been shown to easily change their function [33]. This enlarges our perspective on the emergence of GCCs, MCCs, PCCs and acyl-CoA carboxylases with respect to previous considerations [9,11]

In the available literature, promiscuous acyl-CoA carboxylases are called ACCases [7], but this name can be easily confused with ACCs that specifically carboxylate acetyl-CoA. Thus, a more explicit name should be given to the whole group including PCC, MCC, GCC and acyl-CoA carboxylase sequences in order to point out their common origin whatever their substrate specificity would have become throughout evolution. Therefore, we propose to call this group XCC, for "any CoA-bearing-substrate carboxylase". XCC enzymes are characterized by two subunit types (one BC-BCCP and one CCT), the specificity and quaternary structure of which would have evolved from one generic biotin-dependent enzyme ancestral to bacteria.

Recent works have reported a BT domain located in between the BC and the BCCP domains of the PCC α-subunit [27]. The BT domain has been proposed to be of major relevance for subunit interaction and even though our sequence similarity searches did not identify unequivocally a BT domain in other XCC enzymes, a region of unknown function could be detected between the BC and BCCP domains of all XCC α-subunits. That region shared low sequence similarity with the PCC BT domain but was predicted to contain a helix structure as in the BT/PT domain. Acyl-CoA carboxylases that lack that characteristic helix have been shown to need an additional accE subunit for normal domain interaction [7,32,49], and we have found that the accE also contains a helix structure. Although functional confirmation is required, these results suggest that this unknown region in XCC might be an essential homologue of the BT domain. Therefore, the accE subunit of some acyl-CoA carboxylases would be a substitute developed in BT-lacking α-subunits.

In summary, the presence-absence analyses and the phylogenies of BC, CCT, PCT and UCT support the ancestral presence of ACC, PYC and XCC in bacteria. In addition, specific functions would have arisen from XCCs by duplication, subfunctionalization and HGT events across bacterial evolution. Nevertheless, the absence of some of these enzymes in several bacterial lineages that have been proposed to have diverged early in the bacterial domain (such as the Thermotogae and Aquificae) opens the possibility that they may have evolved after the last common ancestor of all Bacteria. Therefore, the analysis of the content of biotin-dependent carboxylases in the other domains of life is necessary to infer whether these enzymes are ancestral or not.

### Biotin-dependent carboxylases in eukaryotes

In most of our biotin-dependent carboxylase phylogenies, eukaryotic sequences branch within their bacterial homologues, what strongly supports HGTs events from bacteria to an ancestor of the eukaryotes as the most likely way for them to have acquired these enzymes. Some of these HGTs probably took place through endosymbiosis, as suggested by the cellular localization of eukaryotic PCCs and MCCs in mitochondria [56,57] and the mitochondria-targeting signal peptides observed in their N-terminal regions. In addition to the typical eukaryotic ACC polypeptide, plastid-bearing eukaryotes also carry a bacterial-like multi-subunit ACC [8] with an N-terminal region containing a plastid-targeting sequence. Contrary to typical eukaryotic ACC polypeptides that form an independent group, these eukaryotic bacterial-like ACCs branch among cyanobacterial ACCs, strongly supporting an endosymbiotic origin (Figure 3).

Targeting sequences seem to be absent from eukaryotic PYC and ACC. Both BC- and PCT-domain phylogenies strongly support the branching of eukaryotic PYC sequences among their bacterial homologues, what indicates a bacterial origin even though the vector of this HGT cannot be specified. Surprisingly, concerning the eukaryotic ACCs, the phylogenetic reconstructions are at odds with protein domain comparison. On the one hand, eukaryotic ACCs branch among bacterial ACC sequences in preliminary CCT and accurate BC phylogenies (additional file 1 and Figure 3, respectively), suggesting that bacterial donors are at the origin to these sequences. However, eukaryotic ACC sequences form extremely divergent groups in both BC and CTT phylogenies, probably as a consequence of rapid evolution subsequent to the fusion of these domains to generate the eukaryotic ACC polypeptide. Thus, reconstruction artifacts are likely and little confidence can be given to the position of eukaryotic sequences in these phylogenies. On the other hand, protein domain composition of eukaryotic ACC is very different from those of PYCs and bacterial ACCs, but shares strong similarity with that of XCC (Figure 2). Huang *et al*. proposed that the most likely way to explain the unique domain composition of eukaryotic ACC is a fusion event between the α- and β-subunits of XCC on both sides of a central linker [27]. The elucidation of the function of the BT/PT domain of eukaryotic ACCs will be necessary to decide between the XCC and the bacterial ACC origin for the eukaryotic ACC polypeptides.

Finally, the BC phylogeny indicates that UCA genes were acquired recently and independently by fungi and green algae from bacteria. Whereas eukaryotes have maintained the same domain structure than their bacterial counterparts for PYC, MCC, PCC and bacterial-type ACC, the components of eukaryotic ACC and UCA have fused to generate large polypeptides.

### Biotin-dependent carboxylases in Archaea

Although recent studies have revealed the importance of biotin-dependent carboxylases in Archaea, little is known about their origin and evolution [28,29,40]. These enzymes are much less abundant and diversified in Archaea than in the other two domains of life, but their study could be of major interest in understanding the emergence and ancient evolution of several central metabolic pathways in these organisms [58]. In Archaea, the BC domain is encoded by an independent subunit in both the PYC and the ACC-PCC complexes, instead of being a part of wider polypeptides. BC sequences are widespread among archaea and their phylogeny shows that major archaeal groups form monophyletic clades, which are more closely related among them than to any other taxon. This evidence supports the ancestral presence of the BC domain in Archaea and points to its vertical inheritance in this domain of life. Surprisingly, archaeal BC sequences do not cluster together with regard to the PYC and ACC-PCC functional groups, but rather following the accepted phylogeny of Archaea. This strongly supports the hypothesis for the presence of one unique promiscuous BC subunit in the last common archaeal ancestor that would have been recruited in both, PYC and ACC-PCC functions. In the other hand, the PCT phylogeny and its distribution in archaea also indicate that the PYC function may have existed in the archaeal common ancestor. Concerning the ACC-PCC function, the wide distribution of the CCT domain in archaea suggests that it could also be ancestral. Moreover, even though archaeal CCT sequences were not found to be monophyletic in our CCT phylogenies, the AU test could not reject the hypothesis of the archaeal monophyly, and thus the presence of a CCT subunit in the archaeal common ancestor can be hypothesized. As a result, one unique promiscuous BC subunit that collaborated with PCT and CCT subunits to catalyze their specific reactions may have existed in the last common archaeal ancestor. Interestingly, such a collaboration of a BC-encoding subunit with both the CCT and PCT systems has been suggested in *Archaeoglobus fulgidus *[59] although, to our knowledge, this has not yet been experimentally tested. Our analyses support to extend this possibility to the ancestor of Archaea.

An alternative origin of archaeal biotin carboxylases through ancient HGTs from bacteria cannot be completely excluded. However, except for some particular cases limited to some specific organisms, the distribution and the phylogenetic trees of the BC and the PCT domains are congruent with the expected phylogeny of Archaea. As a result, the hypothetical HGT events responsible for the presence of the BC and PCT domains in Archaea should have been ancient enough to predate the last common archaeal ancestor, and thus HGT cannot be favored over the simple vertical inheritance from the cenancestor. Concerning the CCT domain, some groups, as for example the Halobacteriales, may have inherited their sequences by HGT from bacterial donors, but our results do not provide enough support neither to the monophyly of all archaeal CCTs nor to the putative HGT-mediated origin of these sequences, so this issue remains open.

Relatively recent HGT events cannot be ignored in the study of the evolutionary history of biotin-dependent carboxylases. They may have played an important role, both in the case of closely related organisms (for example, HGTs of XCC among Actinobacteria), as well as for distantly related taxa (e.g., the archaeal-type PYC transferred to some aquificales and epsilon-proteobacterial species). HGTs may have also been at the origin of completely new functions. For instance, bacterial ODC has been recently proposed to use one decarboxylating subunit common to all decarboxylases and a CT domain from any other origin [60]. ODC-CT is restricted to a limited number of anaerobic bacteria and is closely related to archaeal PCT, what supports the archaeal origin of ODC-CT. Possible criticism against this HGT hypothesis could be that the bacterial ODC sequences do not branch within the archaeal PCT sequences but as a sister group (Figure 6). Nevertheless, the divergence of the bacterial sequences in order to acquire their new function is likely to have induced a long branch attraction artifact in this phylogeny.

### Occurrence of BCCP: BPL-BirA evolution in the three domains of life

Contrarily to the rest of the protein domains involved in biotin-dependent carboxylation, we did not analyzed the evolution of the BCCP domain because of the limited phylogenetic signal due to its small size (~ 90 amino acids) and low sequence conservation, which make the BCCP phylogenies very sensitive to reconstruction artifacts. In addition, this domain can be found in many different polypeptide architectures, including transcarboxylases and decarboxylases that are not part of our work. A phylogenetic analysis of BCCPs published some years ago [59] supported the occurrence of many different fusion and fission events with the PCT domains and only one fusion event with the BC domain in the XCC sequences. Such complex fusion and fission scenario should be balanced by the recent discovery of the PT domains in PYC sequences [36], which makes independent fusions much less parsimonious than previously suggested.

We analyzed the biotin protein ligase (BPL) protein domain instead of the BCCP domain itself in order to reconstruct a more reliable phylogeny that could provide indirect evidence of the BCCP content in the cenancestor. BPL is related to the lipoyl protein ligases [21] and is responsible for the specific biotinylation of BCCP. In prokaryotes, some species bear a BPL gene, whereas others contain BirA genes. BirA proteins consist of a transcriptional regulation domain in the N-terminus and a BPL domain in the C-terminus. Because BirA could be found in both bacteria and archaea, it has been proposed to be the most ancient known transcriptional factor, already present in the cenancestor [22]. Nevertheless, universal phylogenetic analyses of BPL and BirA had never been carried out to test this hypothesis.

Our results confirmed previous observations of single BPL or BirA genes in each species. Except for some likely HGTs events, our phylogenetic reconstructions of the BPL protein domain showed a phylogeny congruent with the main accepted taxonomic groups in the three domains of life, suggesting a dominant vertical inheritance from the common ancestor to all living organisms (Figure 7). The most puzzling feature of this phylogeny was the distribution of the sequences bearing the N-terminal transcriptional regulation domain characteristic of BirA all over the prokaryotic part of the BPL tree and mixed up with the sequences lacking that domain. This observation could be explained either by an ancestral BPL gene that would have fused many independent times with the N-terminal transcriptional regulator to form different BirA genes, or by an ancestral BirA gene that would have lost its N-terminal regulatory domain in many lineages independently. As the latter hypothesis appears more parsimonious than the former, our results tend to support the BirA gene to be ancestral to all organisms, as previously proposed [22]. Finally, this evidence supporting the presence of the BPL function in the cenancestor is an additional indirect argument in favor of the presence of the BCCP domain proteins in this ancestral organism.

## Conclusions

To summarize, our data support that ACC, XCC and PYC are ancestral to Bacteria. MCC, PCC, GCC and UCA emerged subsequently from XCC in the bacterial lineage. Eukaryotes most likely inherited their biotin-dependent carboxylases through different HGTs from bacteria: MCC and PCC are related to alphaproteobacterial homologues and therefore seem to have been acquired through the mitochondrial endosymbiosis, whereas plant bacterial-like ACC is related to cyanobacterial homologues and thus of probable plastid origin. The remaining eukaryotic enzymes (ACC, PYC and UCA) have likely been also transferred from bacteria but their donors are unknown. The last common archaeal ancestor likely used a promiscuous BC subunit with PCT and CCT subunits to ensure PYC and ACC/PCC functions, respectively. Finally, the cenancestor likely bore a BirA gene able to biotinylate BCCPs. We used these results to infer the set of biotin-dependent carboxylases present in the cenancestor. It is important to note here that the precise nature of the cenancestor remains debated, in particular whether it was a single organism or a community of more or less related different organisms experiencing a high frequency of HGT [61,62]. When we infer that a particular biotin-dependent carboxylase may have been ancestral, this is applicable to both views.

Since eukaryotes obtained their biotin-dependent carboxylases from bacteria, we ignore them for the discussion concerning the cenancestor complement and we focus specifically on the respective ancestors of Archaea and Bacteria as intermediate steps between present-day species and the cenancestor. The components of biotin-dependent carboxylases have been duplicated, recombined and fused many times across evolution and, thus, many different evolutionary scenarios can be proposed. As it would be too long to discuss all of them, we will focus only on the one that we consider to be the most parsimonious (for examples of other scenarios see additional file 3).

**Figure 8:**
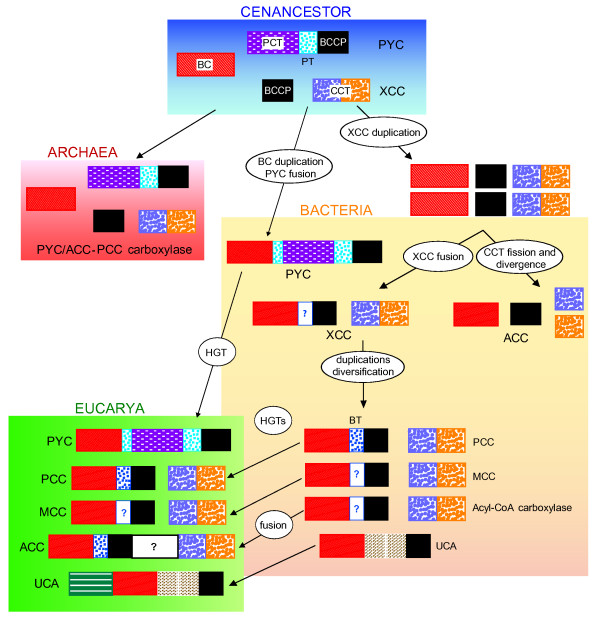
**Evolutionary hypothesis for the early diversification of the biotin-dependent carboxylases**. Colored blocks represent functional protein domains. A biotin-dependent carboxylase content similar to that of modern archaea was present in the cenancestor. It was conserved in Archaea but in Bacteria these enzymes were duplicated and fused several times, bearing to the very diverse modern biotin-dependent carboxylase family found in these organisms. Eukaryotes received their enzymes by horizontal gene transfer (HGT).

In this hypothesis (Figure 8), the cenancestor would have had a relatively simple biotin-dependent carboxylase content similar to that of modern archaea: one promiscuous BC-bearing subunit would have interacted with a PCT-PT-BCCP subunit to catalyze the PYC function and also with independent BCCP and CCT subunits to carry out the carboxylation of CoA-substrates. Ancient archaea would have inherited, conserved and adapted this content to their low biotinylating needs, keeping one PYC and one ACC-PCC, either conserved or lost across subsequent evolution of the different archaeal phyla. In the bacterial lineage, the duplication of the BC domain allowed the emergence of two different biotin-dependent carboxylases, a polypeptidic PYC and one ancient CoA-substrate carboxylase made up of three subunits. The polypeptidic PYC was vertically inherited in bacteria whereas a duplication of the three-subunit CoA-substrate carboxylase took place before the last common bacterial ancestor. One of the resulting CoA-substrate carboxylases had its CCT subunit split in two parts that became the two CCT subunits in the bacterial ACCs. In the other three-subunit enzyme there was a fusion between the BC and BCCP domains to give rise to an ancestral promiscuous XCC. Later duplications and subfunctionalizations lead to the emergence of the very diverse XCC family that we know in contemporary bacteria.

A strong point of this scenario is its relative simplicity, relying on the general assumption of aggregative peptide domain architecture as a major force in protein evolution [63]. Noteworthy, this hypothesis assumes the independent emergence of the PT (ancient PYC) domain and the BT (bacterial XCC) domain. Thus, a convergent evolution has to be invoked to explain BT and PT structural resemblances. Despite that little is known concerning the characteristics and conservation of the BT/PT domains, their shared structure consisting of a conserved helix and several β-strands seems simple enough to hypothesize that it could have emerged twice independently and their conserved position between the BC and the BCCP domains could be the result of structural constraints related to their common subunit-interaction roles.

Although some other scenarios could be proposed based on the results of our phylogenetic and protein domain composition analyses, all of them support the presence of a relatively complex biotin-dependent carboxylase complement in the cenancestor. It would have been able to carboxylate the pyruvate and several CoA-linked substrates. Contemporary biotin-dependent carboxylases contribute to very diverse major biological functions, such as fatty acid biosynthesis, anaplerosis, gluconeogenesis, and autotrophic CO_2 _fixation. Therefore, identifying the precise activity of the ancient biotin-dependent carboxylases proposed to have been present in the cenancestor is a very difficult question. Phylogenetic analyses alone do not provide enough information to clarify this issue. A promising approach would be the bioinformatic inference of the sequences of the ancestral enzymes to synthesize them in order to characterize their activity in detail (what is often called "enzyme resurrection" [64-66]). That would be particularly interesting to check for potential activities involved in fatty acid biosynthesis or autotrophic CO_2 _fixation since the presence of these metabolic pathways in the cenancestor remains a hotly debated question [67-70].

## Methods

### Sequence retrieval and alignment

For each domain of life for which sequence data was available, we retrieved one representative of each of the different biotin-dependent carboxylases and BPL/BirA enzymes from the KEGG database (http://www.genome.jp/kegg) to be used as seeds for further similarity searches. Since some biotin-dependent carboxylases were absent from this database, we completed with archaeal ACC/PCC [29], bacterial GCC [9], one bacterial UCA [44] and one proteobacterial ODC [60] sequence obtained from GenBank (http://www.ncbi.nlm.nih.gov/Genbank). Similarity searches with BLASTp [71] were done with the well-characterized protein domains contained in these representative sequences as queries against their respective domain of life. In cases where a particular enzyme was missing in KEGG for one domain of life, we used sequences from the other domains as queries. Similarity searches in archaea and bacteria were done against a list of completely sequenced genomes available in GenBank (298 bacteria and 55 archaea, additional file 4). In eukaryotes, all searches were done against the complete non-redundant (nr) eukaryote-annotated GenBank database.

Sequences for each protein domain found by these searches in the three domains of life were aligned with Muscle 3.6 [72] or MAFFT v6.814c-b [73]. Alignments were edited with the program ED of the MUST package [74] and redundant and partial sequences were removed at this step. Ambiguously aligned regions were removed prior to phylogenetic analyses using the NET program from the MUST package. Alignments are available in Nexus format as additional files 5, 6, 7, 8 and 9. Preliminary secondary structure searches on MCC, GCC, PYC, XCC and accE (see results) were carried out using APSSP (Advanced Protein Secondary Structure Prediction Server, http://imtech.res.in/raghava/apssp/) and GOR4 [75].

### Phylogenetic analyses

Preliminary trees based on the complete sequence dataset for each enzyme were constructed by the approximately maximum likelihood approach with FastTree 2.1.3 [76] in order to classify sequences in functional classes with respect to well-characterized proteins (see additional file 1). Neighbor joining trees (NJ) [77] using the MUST package [74] were also reconstructed to select representative sequences with which carrying out more detailed maximum likelihood (ML) and Bayesian inference (BI) phylogenetic analyses. ML tree reconstructions were done with the program TREEFINDER [78] with the LG + Γ model [79] and 4 rate categories, which was selected as the best-fit model for all our datasets by the model selection tool implemented in TREEFINDER [78]. Node support was assessed by 1,000 bootstrap replicates with the same model. BI trees were reconstructed using the program MrBayes v. 3.0b4 [80] with a mixed substitution model and a Γ distribution of substitution rates with 4 categories. Searches were run with 4 chains of 1,000,000 generations for which the first 2,500 generations were discarded as "burn in", trees being sampled every 100 generations. Stabilization of the chain parameters was verified using the program TRACER [81]. Approximately unbiased tests [82] were carried out using the test tool implemented in TREEFINDER [78].

## Authors' contributions

JL and DM designed research; JL carried out phylogenetic analyses, and JL and DM wrote the manuscript. All authors read and approved the final version.

## Supplementary Material

Additional file 1 **Schematic phylogenetic trees of biotin carboxylase (BC) sequences and CoA-substrate related carboxyl transferase (CCT) sequences**. These preliminary FastTree reconstructions allowed the functional assignment of each sequence according to a phylogenetic framework and functional data from the available literature. Click here for file

Additional file 2 **Biotin-dependent carboxylase content in bacterial complete genome sequences**. This is an Excel file showing the presence or absence of homologues of the different biotin-dependent carboxylases in a collection of complete genome sequences. Click here for file

Additional file 3 **Alternative evolutionary scenarios for the early diversification of the biotin-dependent carboxylases**. Colored blocks represent functional protein domains. The first scenario postulates the duplication of an ancient biotin-dependent carboxylase and the insertion of a pyruvate carboxylase carboxyl transferase (PCT) within the BT/PT domain predated the cenancestor. This organism had a polypeptidic PYC and a two-subunit XCC that evolved through several duplications and splits in Archaea and Bacteria. The second scenario explores the possibility that the cenancestor bore a CCT divided in two subunits as in contemporary bacterial ACC. Click here for file

Additional file 4 **List of all complete genome sequences used in this work**. This is a text file containing the complete list of species upon which we carried out sequence similarity searches to detect biotin-dependent carboxylase homologues. Click here for file

Additional file 5 **Masked multiple sequence alignment of biotin carboxylase (BC) domain sequences (NEXUS format)**. This alignment was used to reconstruct the tree shown in Figure 3. Click here for file

Additional file 6 **Masked multiple sequence alignment of archaeal and bacterial biotin carboxylase (BC) domain sequences (NEXUS format)**. This alignment was used to reconstruct the tree shown in Figure 4. Click here for file

Additional file 7 **Masked multiple sequence alignment of CoA-substrate related carboxyl tranferase (CCT) domain sequences (NEXUS format)**. This alignment was used to reconstruct the tree shown in Figure 5. Click here for file

Additional file 8 **Masked multiple sequence alignment of pyruvate carboxylase carboxyl tranferase (PCT) domain sequences (NEXUS format)**. This alignment was used to reconstruct the tree shown in Figure 6. Click here for file

Additional file 9 **Masked multiple sequence alignment of biotin carboxyl ligase (BPL) sequences (NEXUS format)**. This alignment was used to reconstruct the tree shown in Figure 7. Click here for file
