# Correction: Early evolution of the biotin-dependent carboxylase family

**DOI:** 10.1186/1471-2148-12-117

**Published:** 2012-07-20

**Authors:** Jonathan Lombard, David Moreira

**Affiliations:** 1 Unité d’Ecologie, Systématique et Evolution, UMR CNRS 8079, Univ. Paris-Sud, Paris-Sud, Orsay Cedex 91405, France

## Correction

After publication of our work [1], we noticed several major mistakes in the figure images provided for final publication: although the main text and the legends are correct, Figure three (Figure 1) has been replaced by an image present in the Addition file 1 and Figure four (Figure 2), Figure five (Figure 3) and Figure six (Figure 4) are displaced with regard to their correct numbers and legends. Please, accept our apologies and refer to the correct corresponding Figure three (Figure 1), Figure four (Figure 2), Figure five (Figure 3) and Figure six (Figure 4) that we provide in this erratum. Legends are the same as in the original article.

**Figure 1:**
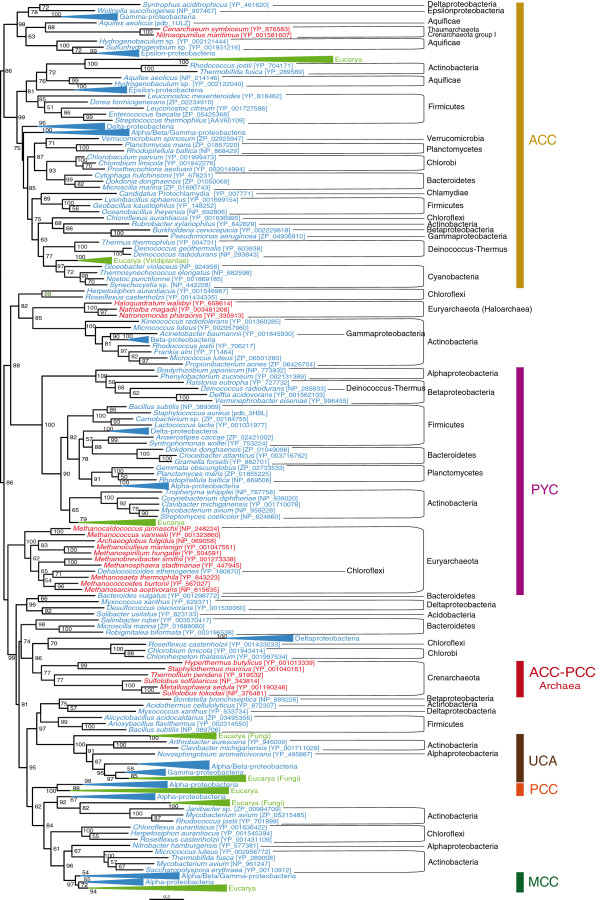
**Maximum likelihood tree of the biotin carboxylase (BC) domain.** This tree is based on 284 representative sequences and 384 conserved sites and was arbitrarily roosted on the bacterial ACC-related sequences. Numbers at nodes indicate bootstrap values higher than 50. Triangles correspond to collapsed groups of eukaryotes and Proteobacteria. Colors on leaves represent the affiliation of the sequences to their respective domain of life: archaea (red), bacteria (blue) and eukaryotes (green). Bars on the right report the functional assignment of the sequences; sequences that are not in front of any bar are assumed to bear an acyl-CoA carboxylase activity.

**Figure 2:**
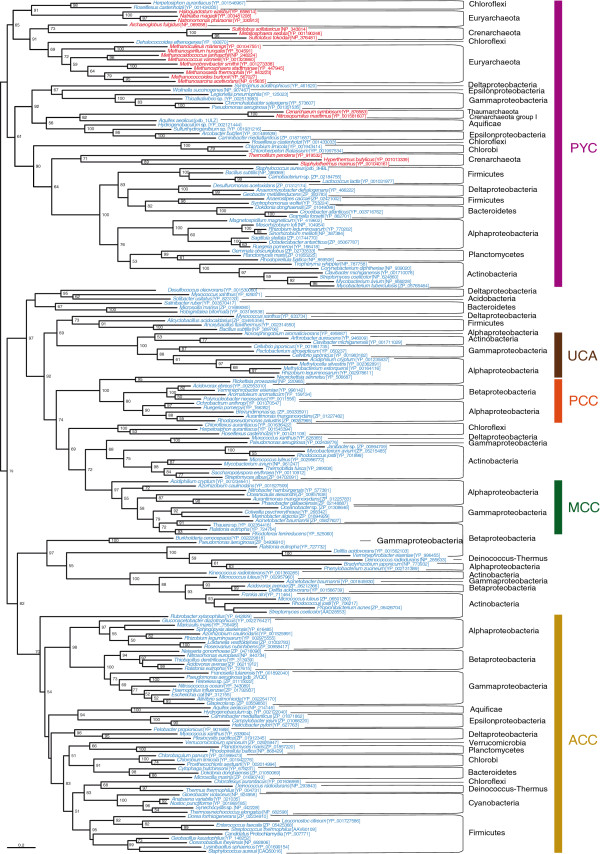
**Maximum likelihood tree of archaeal and bacterial biotin carboxylase (BC) domain sequences.** This tree is based on 196 representative sequences and 322 conserved sites and was arbitrarily rooted on the PYC-related sequences. Numbers at nodes indicate bootstrap robustness values higher than 50. Colors on leaves represent the affiliation of the sequences to their respective domain of life: archaea (red), bacteria (blue) and eukaryotes (green). Bars on the right report the functional assignment of the sequences; sequences that are not in front of any bar are assumed to bear an acyl-CoA carboxylase activity.

**Figure 3:**
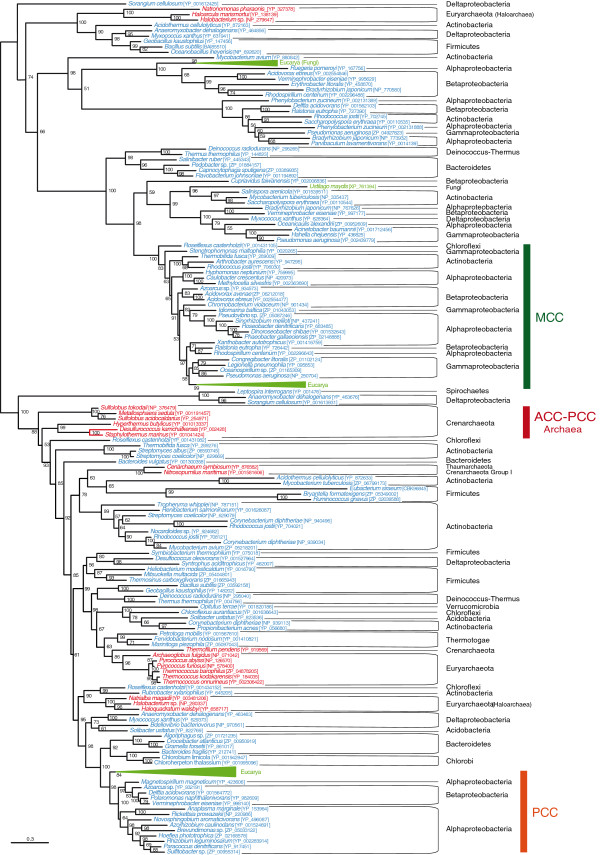
**Maximum likelihood tree of CoA-substrate related carboxyl tranferase (CCT) domain sequences.** This tree is based on 179 representative sequences and 438 conserved sites and was midpoint rooted. Numbers at nodes indicate bootstrap robustness values higher than 50. Triangles correspond to collapsed groups of eukaryotes. Colors on leaves represent the affiliation of the sequences to their respective domain of life: archaea (red), bacteria (blue) and eukaryotes (green). Bars on the right report the functional assignment of the sequences; sequences that are not in front of any bar are assumed to bear an acyl-CoA carboxylase activity.

**Figure 4:**
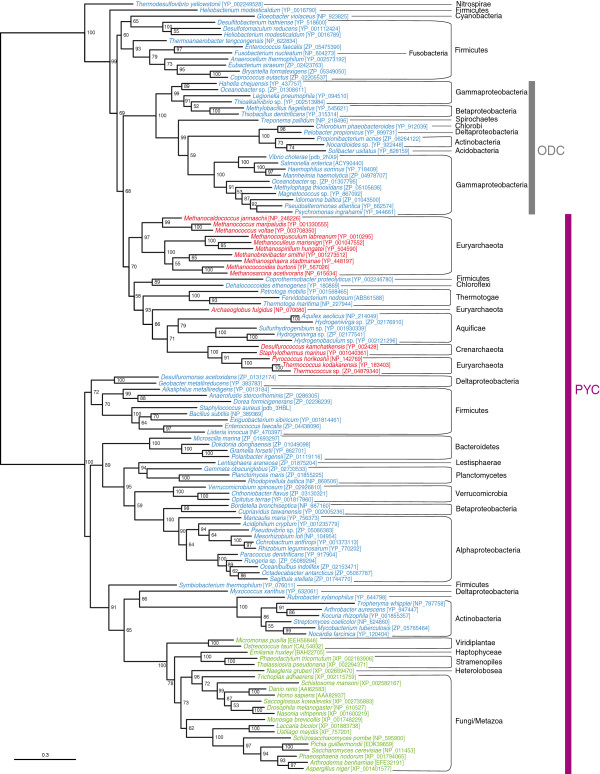
**Maximum likelihood tree of pyruvate carboxylase carboxyl tranferase (PCT) domain sequences.** This tree is based on 126 representative sequences and 432 conserved sites and was midpoint rooted. Numbers at nodes indicate bootstrap robustness values higher than 50. Colors on leaves represent the affiliation of the sequences to their respective domain of life: archaea (red), bacteria (blue) and eukaryotes (green). Bars on the right report the functional assignment of the sequences; sequences that are not in front of any bar have unknown function.

## Competing interests

The authors declare no competing interests.

## Authors' contributions

JL and DM designed research; JL carried out phylogenetic analyses, and JL and DM wrote the manuscript. Both authors read and approved the final version.
