# Supplementary material for: Early evolution of the biotin-dependent carboxylase family
Source: BMC Evol Biol. 2011 Aug 9;11:232. doi: 10.1186/1471-2148-11-232 (PMC3199775; doi:10.1186/1471-2148-11-232)
Supplement: Additional file 1 — Schematic phylogenetic trees of biotin carboxylase (BC) sequences and CoA-substrate related carboxyl transferase (CCT) sequences. These preliminary FastTree reconstructions allowed the functional assignment of each sequence according to a phylogenetic framework and functional data from the available literature. [file 1471-2148-11-232-S1.PDF]

Preliminary phylogenetic analysis of the BC domain

We constructed a first preliminary tree of the biotin carboxylase (BC) domain sequences. It showed different clades corresponding to the diverse substrate specificities of each biotin-dependent carboxylase (data not shown). The eukaryotic ACC biotin carboxylases grouped within one clade clearly separated from the rest of the tree by a long branch, which probably reflected an increased divergence induced by the fusion of BC in a single ACC polypeptide in eukaryotes. Since these very divergent sequences could introduce noise in the phylogenetic reconstruction, we decided to withdraw the polypeptidic ACC eukaryotic sequences to recalculate a new tree. The resulting topology (see Figure A below) showed several clades that we assigned to the functions ACC, PYC, MCC, UCA and one group of PCCs using well-characterized enzymes as reference. This tree was used to classify the BC sequences into these functional groups according to their phylogenetic position. An important number of sequences including the acyl-CoA carboxylases, the GCC and some sequences known to carry out a PCC function but that could also carboxylate other substrates, were not specifically clustered together into unique monophyletic-monofunctional groups. They instead formed a largely unresolved paraphyletic assemblage of deep branches (black branches of the tree), from which the other well-defined functional groups branched off.

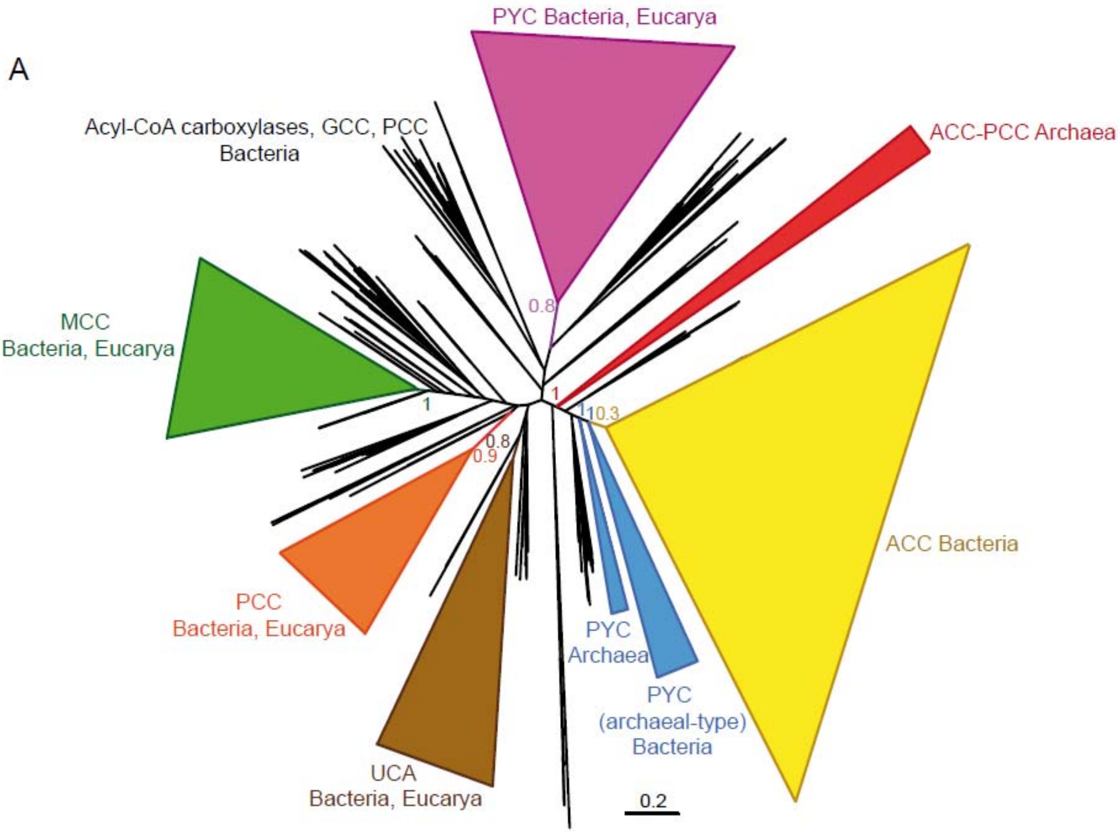

(A) Biotin carboxylase tree reconstructed with the approximately ML FastTree program after removal of eukaryotic ACC-BC sequences. This tree was reconstructed using 1099 sequences and 403 conserved sites and allowed us to classify the BC sequences into different functional groups according to their phylogenetic position.

Preliminary phylogenetic analysis of the CT domains: The CoA-related carboxyl transferase (CCT)

A first preliminary tree containing a large taxonomic sampling (see Figure B below) showed that the two types of bacterial ACC subunits were extremely divergent with regard to the rest of the CCTs, as expected from the very low sequence conservation and the small size of these sequences as observed in multiple alignments. This tree was used to complete a presence-absence pattern of the two CCT subunits of the bacterial ACC (Table 1). After removing these sequences, as well as the eukaryotic ACC sequences that were also extremely divergent and could introduce errors in the phylogenetic reconstruction, a new alignment and tree reconstruction were carried out for the remaining sequences. Functional assignment was then attributed to sequences according to their position in the new tree (data not shown). As for the BC phylogenies, the CCT phylogeny showed several well functionally defined groups (MCC, PCC and archaeal ACC-PCC) and a paraphyletic group made up of the acyl-CoA carboxylases, GCC, some PCC (black branches in the tree).

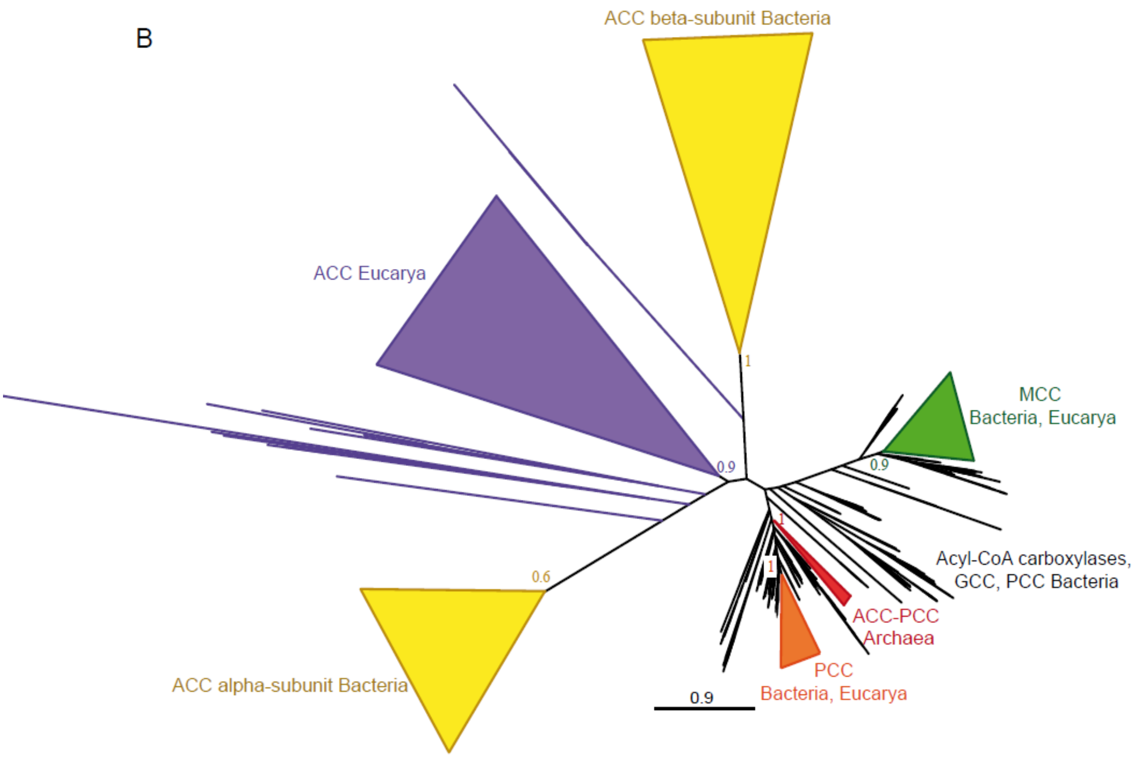

(B) CoA-substrate related carboxyl transferase tree reconstructed with 1075 sequences and 295 conserved sites, it was used for the assignment of the bacterial ACC-CT subunits to their respective groups.
