# Supplementary material for: Early evolution of the biotin-dependent carboxylase family
Source: BMC Evol Biol. 2011 Aug 9;11:232. doi: 10.1186/1471-2148-11-232 (PMC3199775; doi:10.1186/1471-2148-11-232)

Alternative scenarios for the early diversification of the biotin-dependent carboxylases.

1- Alternative scenario 1: In this scenario, the structural and functional similarities between the BT and PT domains as well as their relative position (always preceded by BC and followed by BCCP in PYC and XCC genes) are considered as evidence for a common origin. Since the PT domain is divided in two parts surrounding the PCT domain in PYC polypeptides, this scenario suggests that the PCT was inserted within a pre-existing PT domain. As a result, the BC-BT/PT-BCCP architecture could be assumed to have predated the bacterial contemporary PYC polypeptidic architecture. Starting from a BC-BT/PT-BCCP original architecture, we could propose a duplication event prior to the cenancestor: one of the two copies kept the BC-BT/PT-BCCP architecture and the XCC function related to the interaction with CCT, whereas the second copy underwent the insertion of the PCT domain within the PT domain and resulted in the PYC polypeptide.

In summary, in this scenario a PYC polypeptide and a 2-subunit XCC were present in the cenancestor, conserved in bacteria and modified in archaea. More precisely, the bacterial lineage maintained the polypeptidic PYC whereas the duplication and diversification of XCC led to new genes, one of which split up and formed the bacterial 4-subunit ACC. Present archaea bear only one BC subunit encoded by an independent gene, what supposes that the BC domains moved away respectively from the inherited PYC and XCC ancient genes and one of them was lost before the last common ancestor of Archaea. This hypothesis reasonably explains the conservation of BT/PT domains among PYCs and XCCs but requires many not testable complex fusion hypotheses prior to the cenancestor and many independent divisions from ancient polypeptides in the archaeal and bacterial lineages, an uncommon feature in protein domain architecture evolution.

Alternative scenario 1 for the early diversification of biotin-dependent carboxylases

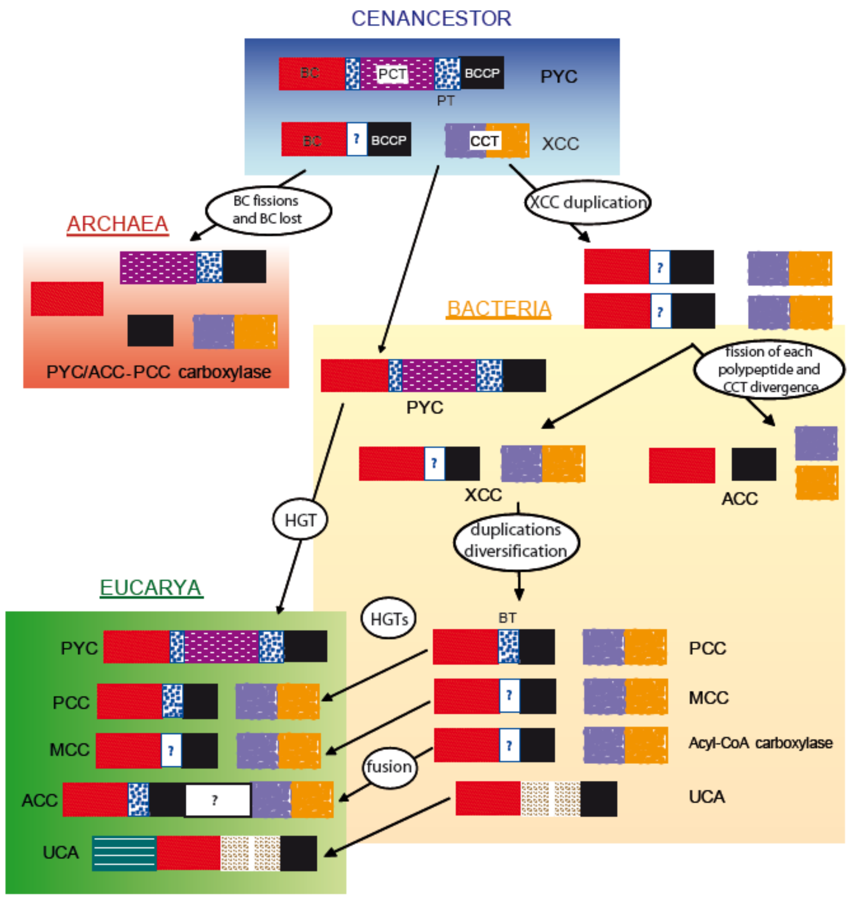

2- Alternative scenario 2:

This scenario specifically addresses the question of the evolution of the CCT domain and can thus be considered both in the context of the scenario presented in the main text and in the context of the additional scenario 1 presented above. In this scenario, we could propose than the cenancestor bore two CCT subunits corresponding to the bacterial CT-ACC subunits. From this ancient CCT content in the cenancestor, the two CCT subunits would have been fused in the archaeal lineage; in the bacterial lineage there would have been one duplication event of the two CT subunits, followed by another fusion event in the origin of the XCC CCT prior to the last common bacterial ancestor. The other two CT subunits remained independent and specialized in the bacterial ACC function.

Alternative scenario 2 for the early diversification of biotin-dependent carboxylases

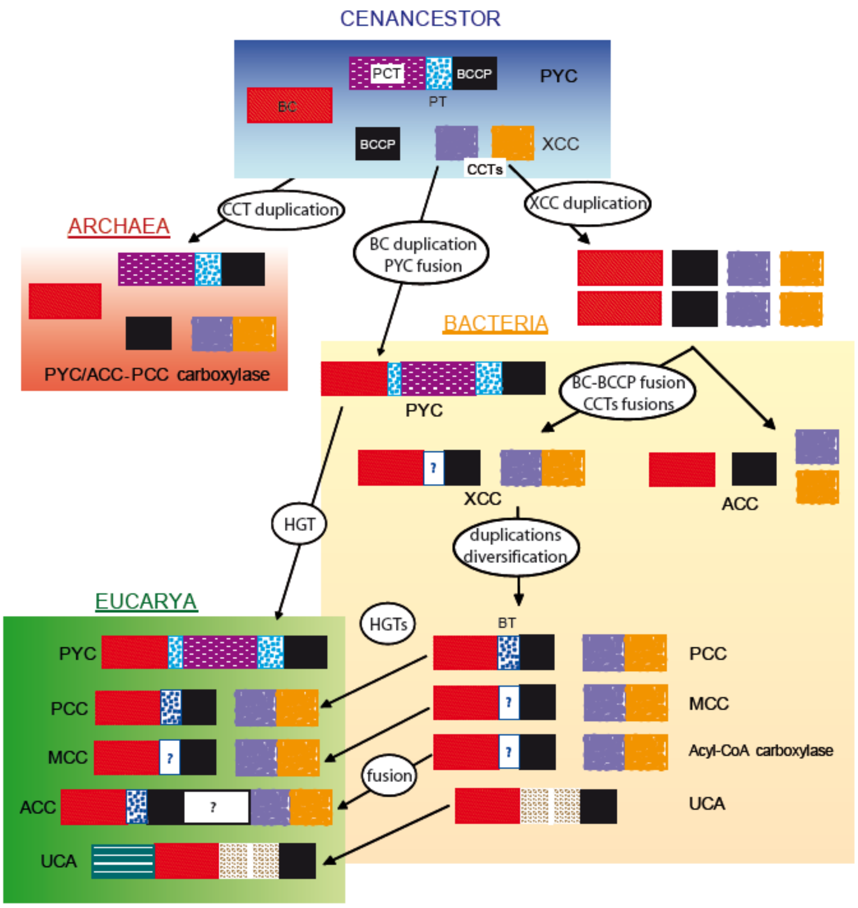

Supplement: Additional file 3 — Alternative evolutionary scenarios for the early diversification of the biotin-dependent carboxylases. Colored blocks represent functional protein domains. The first scenario postulates the duplication of an ancient biotin-dependent carboxylase and the insertion of a pyruvate carboxylase carboxyl transferase (PCT) within the BT/PT domain predated the cenancestor. This organism had a polypeptidic PYC and a two-subunit XCC that evolved through several duplications and splits in Archaea and Bacteria. The second scenario explores the possibility that the cenancestor bore a CCT divided in two subunits as in contemporary bacterial ACC. [file 1471-2148-11-232-S3.PDF]
