# Supplementary material for: Early evolution of the biotin-dependent carboxylase family
Source: BMC Evol Biol. 2011 Aug 9;11:232. doi: 10.1186/1471-2148-11-232 (PMC3199775; doi:10.1186/1471-2148-11-232)
Supplement: Additional file 4 — List of all complete genome sequences used in this work. This is a text file containing the complete list of species upon which we carried out sequence similarity searches to detect biotin-dependent carboxylase homologues. [file 1471-2148-11-232-S4.PDF]

Bacteria

Acidothermus cellulolyticus
Actinomyces odontolyticus
Arthrobacteraurescens
Bifidobacterium adolescentis
Bifidobacterium bifidum
Brevibacterium linens
Clavibacter michiganensis
Collinsella aerofaciens
Corynebacterium diphtheriae
Frankia alni ACN14a
Janibacter sp. HTCC2649
Kineococcus radiotolerans
Kocuria rhizophila
Leifsonia xyli
Micrococcus luteus
Mycobacterium avium
Mycobacterium tuberculosis
Nocardia farcinica
Nocardioides sp. JS614
Propionibacterium acnes
Renibacterium salmoninarum
Rhodococcus jostii
Rubrobacter xylanophilus
Saccharopolyspora erythraea
Salinispora arenicola
Streptomyces albus
Streptomyces coelicolor
Thermobifida fusca
Tropheryma whipplei
Algoriphagus sp. PR1
Alistipes putredinis
Bacteroides fragilis
Bacteroides vulgatus
Capnocytophaga sputigena Capno
Chlorobaculum parvum
Chlorobium limicola
Chlorobium phaeobacteroides
Chloroherpeton thalassium
Croceibacter atlanticus
Cytophaga hutchinsonii
Dokdonia donghaensis
Flavobacterium johnsoniae
Gramella forsetii
Kordia algicida
Leeuwenhoekiella blandensis
Microscilla marina
Parabacteroides johnsonii
Pedobacter sp. BAL39
Pelodictyon luteolum
Polaribacter irgensii
Porphyromonas gingivalis
Prosthecochloris aestuarii
Psychroflexus torquis
Robiginitalea biformata
Salinibacter ruber
Protochlamydia amoebophila
Chlamydia trachomatis
Chlamydophila pneumoniae
Acaryochloris marina
Anabaena variabilis
Arthrospira maxima
Crocosphaera watsonii
Cyanobium sp.
Cyanothece sp. ATCC 51142
Gloeobacter violaceus
Leptolyngbya valderiana
Lyngbya sp. PCC 8106
Microcoleus chthonoplastes
Microcystis aeruginosa
Nodularia spumigena
Nostoc punctiforme PCC 73102
Prochlorococcus marinus
Synechococcus elongatus
Synechocystis sp. PCC 6803
Thermosynechococcus elongatus
Trichodesmium erythraeum
Alicyclobacillus acidocaldarius
Anoxybacillus flavithermus WK1
Bacillus cereus
Bacillus subtilis
Exiguobacterium sibiricum
Geobacillus kaustophilus
Listeria innocua
Lysinibacillus sphaericus
Oceanobacillus iheyensis
Paenibacillus larvae
Pasteuria nishizawae
Staphylococcus aureus
Alkaliphilus metalliredigens
Anaerocellum thermophilum
Anaerococcus hydrogenalis
Anaerofustis stercorihominis
Anaerostipes caccae
Anaerotruncus colihominis
Blautia hansenii
Bryantella formatexigens
Caldicellulosiruptor saccharolyticus
Candidatus Desulforudis audaxviator
Carboxydibrachium pacificum
Carboxydothermus hydrogenoformans
Clostridium acetobutylicum
Clostridium botulinum
Coprococcus eutactus
Coprothermobacter proteolyticus
Desulfitobacterium hafniense
Desulfotomaculum reducens
Dorea formicigenerans
Epulopiscium sp.

Eubacterium siraeum
Faecalibacterium prausnitzii
Finegoldia magna
Halothermothrix orenii
Heliobacterium modesticaldum
Mitsuokella multacida
Moorella thermoacetica
Natranaerobius thermophilus
Parvimonas micra
Pelotomaculum thermopropionicum
Roseburia intestinalis
Ruminococcus gnavus
Symbiobacterium thermophilum
Syntrophomonas wolfei
Thermoanaerobacter tengcongensis
Thermosinus carboxydivorans
Carnobacterium sp. AT7
Enterococcus faecalis
Lactobacillus acidophilus NCFM
Lactococcus lactis
Leuconostoc citreum KM20
Leuconostoc mesenteroides
Oenococcus oeni
Pediococcus pentosaceus
Streptococcus agalactiae
Streptococcus thermophilus
Acholeplasma laidlawii
Acidobacteria bacterium Ellin345
Akkermansia muciniphila
Aquifex aeolicus
Blastopirellula marina
Chloroflexus aurantiacus
Chthoniobacter flavus
Dehalococcoides ethenogenes
Deinococcus geothermalis
Deinococcus radiodurans
Dictyoglomus thermophilum
Elusimicrobium minutum
Fervidobacterium nodosum
Fusobacterium nucleatum
Gemmata obscuriglobus
Herpetosiphon aurantiacus
Hydrogenivirga sp. 128-5-R1-1
Hydrogenobaculum sp. Y04AAS1
Lentisphaera araneosa HTCC2155
Marinitoga piezophila KA3
Mesoplasma florum L1
Methylacidiphilum infernorum V4
Mycoplasma genitalium G37
Mycoplasma pneumoniae M129
Mycoplasma pulmonis UAB CTIP
Opitutus terrae PB90-1
Petrotoga mobilis SJ95
Planctomyces maris DSM 8797
Rhodopirellula baltica SH 1
Roseiflexus castenholzii DSM 13941
Solibacter usitatus Ellin6076
Sulfurihydrogenibium sp. YO3AOP1
Thermodesulfovibrio yellowstonii DSM 11347
Thermosipho africanus TCF52B
Thermotoga maritima MSB8
Thermus thermophilus
Verrucomicrobium spinosum DSM 4136
Victivallis vadensis
bacterium Ellin514
Magnetococcus sp. MC-1
Mariprofundus ferrooxydans PV-1
Acidiphilium cryptum JF-5
Aurantimonas sp. SI85-9A1
Azorhizobium caulinodans ORS 571
Bartonella henselae str. Houston-1
Beijerinckia indica
Bradyrhizobium japonicum USDA 110
Brevundimonas sp. BAL3
Brucella abortus
Caulobacter crescentus CB15
Dinoroseobacter shibae DFL12
Erythrobacter litoralis HTCC2594
Fulvimarina pelagi HTCC2506
Gluconacetobacter diazotrophicus PAl 5
Gluconobacter oxydans 621H
Granulibacter bethesdensis CGDNIH1
Hoeflea phototrophica DFL-43
Hyphomonas neptunium ATCC 15444
Jannaschia sp. CCS1
Labrenzia aggregata IAM 12614
Loktanella vestfoldensis SKA53
Magnetospirillum magneticum AMB-1
Maricaulis maris MCS10
Mesorhizobium loti MAFF303099
Methylobacterium extorquens PA1
Methylocella silvestris BL2
Nitrobacter hamburgensis X14
Novosphingobium aromaticivorans DSM12444
Oceanibulbus indolifex HEL-45
Oceanicaulis alexandrii HTCC2633
Oceanicola batsensis HTCC2597
Ochrobactrum anthropi ATCC 49188
Octadecabacter antarcticus
Oligotropha carboxidovorans OM5
Paracoccus denitrificans PD1222
Parvibaculum lavamentivorans DS-1
Parvularcula bermudensis HTCC2503
Phaeobacter gallaeciensis
Phenylobacterium zucineum HLK1
Pseudovibrio sp. JE062
Rhodobacter sphaeroides
Rhodopseudomonas palustris
Rhodospirillum centenum SW
Roseobacter denitrificans OCh 114

Roseovarius nubinhibens ISM
Ruegeria sp. R11
Sagittula stellata E-37
Silicibacter pomeroyi DSS-3
Sphingomonas wittichii RW1
Sphingopyxis alaskensis RB2256
Sulfitobacter sp. EE-36
Xanthobacter autotrophicus Py2
Zymomonas mobilis subsp. mobilis ZM4
Agrobacterium tumefaciens str. C58
Rhizobium leguminosarum
Sinorhizobium meliloti 1021
Anaplasma marginale str. St. Maries
Candidatus Pelagibacter ubique
Ehrlichia canis str. Jake
Neorickettsia sennetsu str. Miyayama
Orientia tsutsugamushi
Rickettsia prowazekii str. Madrid E
Bordetella bronchiseptica RB50
Burkholderia cenocepacia
Cupriavidus taiwanensis
Limnobacter sp. MED105
Polynucleobacter necessarius
Ralstonia eutropha H16
Chromobacterium violaceum ATCC12472
Neisseria gonorrhoeae
Acidovorax avenae
Aromatoleum aromaticum EbN1
Azoarcus sp. BH72
Comamonas testosterone KF-1
Dechloromonas aromatica RCB
Delftia acidovorans SPH-1
Diaphorobacter sp. TPSY
Herminiimonas arsenicoxydans
Janthinobacterium sp. Marseille
Leptothrix cholodnii SP-6
Methylibium petroleiphilum PM1
Methylobacillus flagellatus KT
Nitrosomonas europaea ATCC 19718
Nitrosospira multiformis ATCC 25196
Polaromonas naphthalenivorans CJ2
Rhodoferax ferrireducens T118
Thauera sp. MZ1T
Thiobacillus denitrificans ATCC 25259
Verminephrobacter eiseniae EF01-2
Anaeromyxobacter dehalogenans
Bdellovibrio bacteriovorus HD100
Desulfatibacillum alkenivorans AK-01
Desulfococcus oleovorans Hxd3
Desulfotalea psychrophila LSv54
Desulfovibrio vulgaris
Desulfuromonas acetoxidans DSM 684
Geobacter metallireducens GS-15
Lawsonia intracellularis PHE/MN1-00
Myxococcus xanthus DK 1622
Pelobacter propionicus DSM 2379
Plesiocystis pacifica SIR-1
Sorangium cellulosum 'So ce 56'
Stigmatella aurantiaca DW4/3-1
Syntrophobacter fumaroxidans MPOB
Syntrophus aciditrophicus SB
Arcobacter butzleri RM4018
Caminibacter mediatlanticus TB-2
Campylobacter jejuni
Helicobacter pylori
Nitratiruptor sp. SB155-2
Sulfurimonas denitrificans DSM1251
Sulfurovum sp. NBC37-1
Wolinella succinogenes DSM 1740
Buchnera aphidicola
Citrobacter koseri ATCC BAA-895
Enterobacter sakazakii ATCC BAA-894
Erwinia tasmaniensis Et1/99
Escherichia coli O157:H7
Klebsiella pneumoniae
Pectobacterium atrosepticum SCRI1043
Photorhabdus luminescens Laumondii TTO1
Proteus mirabilis HI4320
Providencia alcalifaciens DSM 30120
Salmonella typhimurium LT2
Shigella flexneri
Sodalis glossinidius str. 'morsitans'
Wigglesworthia glossinidia
Acidithiobacillus ferrooxidans
Acinetobacter baumannii
Aeromonas hydrophila ATCC 7966
Alcanivorax borkumensis SK2
Alkalilimnicola ehrlichei MLHE-1
Alteromonas macleodii
Baumannia cicadellinicola
Beggiatoa sp. PS
Candidatus Carsonella ruddii PV
Candidatus Ruthia magnifica
Candidatus Vesicomyosocius okutanii HA
Chromohalobacter salexigens DSM 3043
Colwellia psychrerythraea 34H
Congregibacter litoralis KT71
Coxiella burnetii
Dichelobacter nodosus VCS1703A
Endoriftia persephone
Francisella tularensis
Glaciecola sp. HTCC2999
Hahella chejuensis KCTC 2396
Halorhodospira halophila SL1
Idiomarina baltica OS145
Legionella pneumophila
Marinobacter algicola DG893
Methylococcus capsulatus str. Bath
Methylophaga sp. DMS010
Moritella sp. PE36

Neptuniibacter caesariensis
Nitrosococcus oceani
Oceanobacter sp. RED65
Pseudoalteromonas atlantica T6c
Psychrobacter arcticus 273-4
Psychromonas ingrahamii 37
Reinekea sp. MED297
Rickettsiella grylli
Saccharophagus degradans 2-40
Shewanella baltica
Thioalkalivibrio sp. HL-EbGR7
Thiomicrospira crunogena XCL-2
Actinobacillus pleuropneumoniae
Haemophilus influenzae
Histophilus somni
Mannheimia haemolytica PHL213
Pasteurella multocida subsp. multocida str.Pm70
Azotobacter vinelandii
Cellvibrio japonicus Ueda107
Pseudomonas aeruginosa
Aliivibrio salmonicida LFI1238
Photobacterium profundum
Vibrio cholerae
Stenotrophomonas maltophilia
Xanthomonas oryzae
Xylella fastidiosa
Borrelia burgdorferi
Leptospira interrogans
Treponema pallidum

Archaea

Aeropyrum pernix K1
Desulfurococcus kamchatkensis 1221n
Hyperthermus butylicus DSM 5456
Ignicoccus hospitalis KIN4/I
Staphylothermus marinus F1
Metallosphaera sedula DSM 5348
Sulfolobus acidocaldarius DSM 639
Sulfolobus solfataricus P2
Sulfolobus tokodaii str. 7
Caldivirga maquilingensis IC-167
Pyrobaculum aerophilum str. IM2
Pyrobaculum arsenaticum DSM 13514
Pyrobaculum calidifontis JCM 11548
Pyrobaculum islandicum DSM 4184
Thermofilum pendens Hrk 5
Thermoproteus neutrophilus V24Sta
Archaeoglobus fulgidus DSM 4304
Haloarcula marismortui ATCC 43049
Halobacterium salinarum
Haloquadratum walsbyi DSM 16790
Halorubrum lacusprofundi ATCC 49239
Natronomonas pharaonis DSM 2160Methanobrevibacter smithii
Methanosphaera stadtmanae DSM 3091
Methanothermobacter thermautotrophicus str. Delta H
Methanocaldococcus jannaschii DSM 2661
Methanococcus aeolicus Nankai-3
Methanococcus maripaludis
Methanococcus vannielii SB
Methanococcus voltae A3
Candidatus Methanoregula boonei 6A8
Methanocorpusculum labreanum Z
Methanoculleus marisnigri JR1
Methanospirillum hungatei JF-1
Methanopyrus kandleri AV19
Methanococcoides burtonii DSM 6242
Methanosaeta thermophila PT
Methanosarcina acetivorans C2A
Methanosarcina barkeri str. Fusaro
Methanosarcina mazei Go1
Pyrococcus abyssi GE5
Pyrococcus furiosus DSM 3638
Pyrococcus horikoshii OT3
Thermococcus barophilus MP
Thermococcus kodakarensis KOD1
Thermococcus onnurineus NA1
Thermococcus sp. AM4
Ferroplasma acidarmanus fer1
Picrophilus torridus DSM 9790
Thermoplasma acidophilum DSM 1728
Thermoplasma volcanium GSS1
Nanoarchaeum equitans Kin4-M
Nitrosopumilus maritimus SCM1
Cenarchaeum symbiosum A
Natrialba magadii ATCC 430
